# Dynamics of the Magnitude, Breadth and Depth of the Antibody Response at Epitope Level Following Dengue Infection

**DOI:** 10.3389/fimmu.2021.686691

**Published:** 2021-07-05

**Authors:** Francesca Falconi-Agapito, Karen Kerkhof, Xiomara Merino, Johan Michiels, Marjan Van Esbroeck, Koen Bartholomeeusen, Michael Talledo, Kevin K. Ariën

**Affiliations:** ^1^ Department of Biomedical Sciences, Unit of Virology, Institute of Tropical Medicine, Antwerp, Belgium; ^2^ Virology Unit, Instituto de Medicina Tropical Alexander von Humboldt, Universidad Peruana Cayetano Heredia, Lima, Peru; ^3^ Department of Clinical Sciences, National Reference Center for Arboviruses, Institute of Tropical Medicine, Antwerp, Belgium; ^4^ Department of Biomedical Sciences, University of Antwerp, Antwerp, Belgium

**Keywords:** peptide microarray, dengue, antibody epitopes, antibody evolution, flavivirus

## Abstract

Dengue is a major public health problem in tropical and sub-tropical regions worldwide. Since the Zika epidemic and the increased co-circulation of other arboviruses, the serology-based diagnosis of dengue has become more problematic due to the high antigenic resemblance, especially among the flavivirus family. Therefore, a more comprehensive understanding of the diversity, specificity and temporal evolution of the antibody response following dengue infection is needed. In order to close this knowledge gap, we used a high-density peptide microarray of 9,072 linear peptides covering the entire proteome diversity of dengue, Zika, yellow fever and chikungunya viruses. The IgM and IgG antibody responses were measured against the designed microarray in symptomatic dengue infected individuals from an arbovirus endemic area in Peru and in overseas travelers returning to Belgium, as representatives of multiple-exposed and primary infections, respectively. Serum samples were collected longitudinally across four time points over the period of six months in Peru and over two time points in travelers. We show that epitopes eliciting the strongest flavivirus cross-reactive antibodies, in both primary and secondary infections were concentrated in the capsid, E, NS1, NS3 and NS5 proteins. The IgG antibody responses against NS1 and NS3 followed a rise-and-fall pattern, with peak titers between two to four weeks after onset of illness. The response to the E and NS5 proteins increased rapidly in the acute phase and was maintained at stable levels until at least 6 months after illness. A more scattered IgM antibody reactivity across the viral proteome was observed in the acute phase of the disease and that persisted through the 6-month window. The magnitude, breadth (i.e. number of unique epitopes targeted) and depth (i.e. number of epitope variants recognized) of the IgG response was higher in secondary infections compared to primary infections. For IgM antibodies, the magnitude of the response was higher in primary infected individuals whereas the breadth and depth of the response was lower in this group compared with the endemic subjects. Finally, through this arboviral proteome-wide epitope mapping, we were able to identify IgM and IgG dengue-specific epitopes which can be useful serological markers for dengue diagnosis and serostatus determination.

## Introduction

Arboviruses represent a large group of viruses transmitted by arthropod vectors, predominantly mosquitoes and ticks. Given their worldwide (re-)emergence in the last decade, they have gained a high priority for global public health (1). In Peru and other tropical regions around the globe, the genera *Flavivirus*, in particular dengue virus (DENV), Zika virus (ZIKV), and yellow fever virus (YFV), and *Alphavirus*, in particular chikungunya virus (CHIKV), are seasonally and geographically widely distributed (2, 3).

Most of the arboviral infections are either asymptomatic or with presentation of mild symptoms including fever, rash or malaise, followed by a rapid resolution of symptoms. However, some patients develop complicated illness after the non-specific phase. These complications include: (i) hemorrhagic fever with DENV and YFV; (ii) congenital disorders associated with ZIKV infection during pregnancy; (iii) encephalitis associated with neuroinvasive viruses such as DENV, West Nile virus (WNV), Saint Louis encephalitis (SLEV); and (iv) severe arthritis following CHIKV infections (1, 4). An early diagnosis and timely management of cases are crucial for disease outcome.

Determining factors for a poor prognosis in the clinical outcome of arboviral diseases are not fully understood. However, it has been well established that pre-existing heterotypic immunity plays a critical role in dengue disease-enhancement. For instance, a subsequent infection with a different DENV serotype represents the greatest risk factor for dengue hemorrhagic fever/dengue shock syndrome (DHF/DSS) (4, 5). Lately, it was also found that prior ZIKV infection enhances subsequent DENV2 infection and increases disease severity (6).

Recent insights have underpinned a critical role played by antibody-dependent enhancement (ADE) as the mechanism leading to the observed higher risk to develop severe forms of disease in subsequent flavivirus infections (4, 6–8). The antigenically related flaviviruses can induce protective type-specific antibodies (TS-Abs) in naïve individuals, but also broadly flavivirus cross-reactive antibodies (CR-Abs). It is presumed that during a subsequent exposure with a different flavivirus, instead of mounting a specific immune response, the infected individuals could elicit an anamnestic humoral response as consequence of the “original antigenic sin” (9, 10). This phenomenon occurs when the immunological memory to highly genetic and structural similar immunogens dominates the response in a subsequent infection, then rendering cross-reactive non-neutralizing Abs. Consequently, exacerbated illness occurs when these pre-existing antibodies instead of neutralizing enhance the active transportation of virus particles into antigen-presenting cells, mainly through fragment crystallizable (Fc) gamma receptors (FcγRs) leading to an increased replication of the virus that finally translates into more severe clinical manifestations of the disease.

Lately, several *in vitro* studies on ADE also reported that cross-reactive West Nile Virus (WNV) Abs enhance ZIKV infection (11, 12) and that ZIKV Abs increase WNV, SLEV, Rocio virus (ROCV) and Ilheus virus (ILHV) infection (13). However, larger clinical studies in flavivirus endemic human populations are needed to establish the role of pre-existing flavivirus humoral responses with either a protective or detrimental role in subsequent flavivirus infections.

This enhancement in developing severe symptoms due to pre-existing heterotypic immunity also extends to vaccines. The only licensed dengue vaccine Dengvaxia^®^ (Sanofi) (CYD-TDV), registered an excess of hospitalizations among seronegative vaccine recipients (14). The main hypothesis for the excess of hospitalized cases is that the vaccine could mimic a primary infection placing the recipient at an increased risk to develop severe dengue in a subsequent infection. In light of this evidence, the World Health Organization (WHO) only recommends its administration to dengue seropositive individuals (15).

Collectively, these studies outline the importance of an early identification of the causative agent of a suspected arboviral case and the need for discerning naïve from experienced flavivirus individuals due to their relevance in a prompt case management and for vaccination strategies (16–19).

In endemic regions the confirmation of arboviral cases relies heavily on the presence of clinical symptoms and the epidemiological context, and not on laboratory results. In the period of 2017-2020, between 20.3%-44.7% of the total dengue cases reported by the Pan-American Health Organization were confirmed by laboratory assays (20). Unfortunately, these reports are often inaccurate due to an increasing co-circulation of flaviviruses in endemic areas and the non-discriminating symptoms developed by patients during the acute phase of the disease (1, 3).

Consequently, reliable diagnosis of arboviral infections require accurate laboratory techniques. The real time RT-PCR (reverse transcriptase) test and/or virus isolation are the most sensitive and specific (21); nevertheless, the limited diagnostic capacity in endemic areas make these tools often unavailable in primary health care centers in Low- and Middle-Income Countries (LMIC). Instead, the detection of antibodies (Abs) or abundantly expressed antigens (e.g. NS1 in DENV and ZIKV) by ELISA or rapid tests is frequently used in resource-limited settings (21, 22). However, several criteria should be taken into consideration when interpreting Ab-based assays.

Firstly, the dynamics of the humoral immune response to arboviruses varies between a primary and secondary infection and this will impact the interpretation of the test results. IgM detection has proved to be useful in diagnosing primo-infected individuals, given its rapid appearance in the acute phase of the disease. However, because IgM can persist longer than 2-3 months after the onset of symptoms (AOS), its detection can be confounded with a recent infection. Therefore, seroconversion in paired samples at different timepoints is often required. In addition, IgM levels in flavivirus-experienced patients are frequently negligible leading to false-negative results (23). IgG Abs, following a different kinetics, become detectable a few days later compared to IgM in primary infections and they can persist for life, while in secondary infections the IgG levels increase rapidly AOS and with higher titers. However, as a consequence of the “original antigenic sin” the rapid raise in IgG does not always correspond to a specific humoral response against the latest infection.

Secondly, the accuracy of serological tests depends on the nature of the antigen used in the assay. Highly similar epitopes known to elicit CR-Abs can confound diagnosis (24). However, given that the genetic and structural similarities in the *Flaviviridae* family varies across the genome depending on the protein region and the viruses in comparison (25), the detection of CR-Abs can be overcome if diagnostic antigens are properly chosen. Therefore, in the last years there has been an effort to replace conventional antigens (e.g. infected cell lines or fixed virus particles), by recombinant viral proteins or protein domains [e.g. non-structural 1 (NS1), E protein, domain III of E (EDIII)]. These second-generation assays show promising results because of improved specificity (26–28), but do not completely resolve the issue of cross-reactivity (25, 29). Probably, this is because individual homologous proteins from different flaviviruses still present discrete conserved immunodominant regions with high similarity that are recognized by CR-Abs (5, 25, 30). Epitope level analysis therefore could have the potential to add several layers of information through the detailed mapping of regions responsible for eliciting type-specific (TS) or CR-Abs.

Synthetic peptide microarrays are an attractive tool to address the cross-reactivity problems seen in the current available diagnostic assays for Flaviviruses. Peptide microarrays allow a high-throughput screening of the Ab reactivities at the epitope resolution across proteins and even entire viral proteomes. When using spatially spotted microarrays with immobilized overlapping peptides, the Ab profile can be better stratified and information about the precise epitopes, that are potentially relevant for diagnosis, prognosis, and surveillance can be obtained (31–34). Moreover, the multiplexing feature of the microarray technology allows the simultaneous displaying of entire peptide libraries from different arboviruses that can be used in longitudinal studies to determine the Ab-response dynamics and the temporal evolution of TS and CR Abs. Especially in the context of co-circulation of arboviruses, the depiction of the Ab response at epitope resolution, would identify seromarker candidates for a more accurate diagnosis of the virus responsible for the most recent infection in individuals with a previous flavivirus exposure either by natural infection or by vaccination.

Here, we designed a high-density peptide microarray containing 9,072 linear epitopes spanning the entire proteome and diversity of DENV, ZIKV, YFV and the co-circulating alphavirus CHIKV. The CHIKV was included in the design due to its local transmission in the Americas, but for purposes of this study, the analysis was focused on the measurement of the Ab diversity against the flaviviruses only. Using this microarray, we characterized the temporal evolution of the IgG and IgM Ab responses in a cohort of dengue infected individuals from Peru and from overseas travelers returning to Belgium, as representatives of secondary/multiple and primary infections, respectively. Through our analysis we were able to identify DENV-specific and flavivirus immunodominant regions of Ab reactivity.

## Materials and Methods

### Study Subjects

#### Endemic Patients

A prospective longitudinal study was carried out between July 2018 and March 2019 in “Santa Gema Hospital (SGH)” in Yurimaguas, a city located at the Alto Amazonas province in the Peruvian Amazon. Patients with acute, undifferentiated febrile illness, with a temperature ≥ 37°C for 7 days or less, together with at least one of the following symptoms: arthralgia, myalgia, head ache or rash, aged between 5 to 65 years old, attending the SGH were included for the study, regardless of gender and ethnicity. Exclusion criteria included febrile patients admitted in the hospital with an identifiable disease (malaria or leptospirosis) or with a fever for more than seven days and individuals younger than 5 and older than 65 years of age. DENV infection was confirmed by RT-PCR as previously described (35) and subsequently serotyped as DENV-2 using an RT-PCR protocol previously reported (36, 37) and confirmed by genome sequencing (38). PCR positive subjects were followed up during a six-months period in which three additional serum samples were obtained. Ten subjects with four follow-up samples were selected to be included in the microarray analysis. Acute samples were obtained between day 1 to 5 AOS and three convalescent samples: early, between day 10 to 28 AOS; mid, between day 65-126 AOS; and late, day 142 to 203 AOS were also obtained. Immunoreactivity was further characterized by measuring neutralizing Abs (NAbs) against the four DENV serotypes, ZIKV, and CHIKV using in house whole virus neutralization tests (39, 40), and against YFV using PRNT (**Table 1**).

**Table 1:** Samples included in the study.

Group	Number	Age (y)/Sex	YFV vaccine	H	DENV serotype	Country*	Sampling days AOS				NT90 DENV				NT90 ZIKV	NT90 CHIKV	PRNT90 YFV
							1^st^	2^nd^	3^rd^	4^th^	DENV1	DENV2	DENV3	DENV4			
Endemic	1	46/F	Y	N	DENV2	Peru	2	17	126	203	1392.8	819.8	>1600	201.8	>1.600	<50	<20
	2	16/M	Y	Y	DENV2	Peru	4	16	118	196	<50	203.2	138.2	<50	< 50	<50	51
	3	31/F	–	Y	DENV2	Peru	0	20	65	142	246.2	451.9	527.8	220.8	81.09	<50	50
	4	20/M	N	Y	DENV2	Peru	5	28	104	181	<50	358.9	125.9	<50	<50	<50	46
	5	28/F	Y	Y	DENV2	Peru	3	21	97	179	210.6	340.1	552.7	257.8	64.5	<50	88
	6	43/F	Y	N	DENV2	Peru	2	19	91	171	1392.8	493.9	962.4	53.4	492.4	<50	98
	7	17/M	N	N	DENV2	Peru	3	13	82	164	116.5	1365.6	224.0	<50	226.9	<50	92
	8	27/M	N	N	DENV2	Peru	3	24	80	159	808.8	266.9	649.8	218.0	>1.600	<50	22
	9	27/M	Y	N	DENV2	Peru	2	10	78	155	<50	129.6	54.1	<50	< 50	<50	>640
	10	44/F	Y	N	DENV2	Peru	4	12	80	157	115.1	928.1	585.6	<50	73	<50	106
Traveler	1	34/F	N	N	DENV1	Thailand	2	10	–	–	–	–	–	–	–	–	<10
	2	45/M	N	N	DENV2	Cuba	6	18	–	–	–	–	–	–	–	–	<10
	3	54/M	N	N	DENV1	Thailand	8	17	–	–	–	–	–	–	–	–	<10

DENV, dengue virus; ZIKV, Zika virus; YFV, yellow fever virus; CHIKV, chikungunya virus.

H, hospitalization; AOS, After onset of symptoms.

Y, yes; N, no, –, no data available.

*In the traveler group it refers to the country where the infection was contracted.

The endemic samples are representative for secondary/multiple arboviral infections based on: (i) Circulation of DENV1-4, ZIKV, CHIKV and Mayaro virus (MAYV) in Yurimaguas; (ii) YFV vaccination is recommended to persons living in the Peruvian Amazon; and (iii) detection of pre-existing NAbs against all DENV serotypes, ZIKV and YFV in most of the samples (**Table 1**).

#### Travelers

These samples were stored samples from travelers consulting the travel clinic at the Institute of Tropical Medicine Antwerp, Belgium, hosting the national reference center for arboviruses. Three patients with a dengue infection confirmed by PCR (two DENV1 and one DENV2) with two follow-up serum samples from the acute (days 5 to 8 AOS) and convalescent (days 10 to 18 AOS) phase of the disease, were selected for the microarray analysis. Samples were negative for the presence of NAbs against YFV using PRNT, besides they were collected in a period where ZIKV was not circulating in the country where the infection was acquired (**Table 1**).

The traveler samples are representative for primary infections since these patients claimed visiting only once an area where arboviruses are prevalent.

#### Negative Samples

Sixteen samples from Antwerp citizens, with no register of visiting arbovirus endemic areas, testing negative for DENV, ZIKV, YFV, tick borne encephalitis virus and CHIKV by virus neutralization tests, were pooled and included in the microarray assay as a non-endemic negative control. One serum sample from a Lima citizen with no register of visiting endemic areas or receiving the yellow fever vaccine, who tested negative to Flavivirus IFAT (Euroimmun, Lübeck, Germany) was included in the microarray assay as an endemic negative control.

### Ethical Clearance

The study was approved by the ethical review boards of the Peruvian University Cayetano Heredia, Peru (Protocol N° 101480), the Institute of Tropical Medicine Antwerp, Belgium (Protocol N° ITG 1304/19) and the University of Antwerp, Belgium (Protocol N° 19/42/477). The study involved analysis of samples collected in the FA4 – AC4 (ITM-DGD) Framework Agreement 4 (2017–2021). Written informed consent was obtained from adults or–in case of minors–from their caretaker. This study was conducted in compliance with the ethical standards of the latest amended Declaration of Helsinki and of the International Conference Harmonization (ICH) guidelines, plus adhering to local laws and regulations.

### Bioinformatic Analysis of DENV, ZIKV, YFV and CHIKV Proteomes to Create the Peptide Library

We developed an arbovirus peptide microarray covering the proteomes of DENV-1, DENV-2, DENV-3, DENV-4, ZIKV, YFV and CHIKV to perform a precise epitope dissection of the immunodominant regions targeted by IgG and IgM Abs. DENV and ZIKV were selected based on the seasonal outbreaks of these viruses in endemic areas, while YFV was included because vaccination is recommended but not compulsory for people traveling or living in the Amazon. The broad antigenic similarities between these viruses could imply a considerable problem for differential diagnosis. As already mentioned, the CHIKV alphavirus was included in the design but its analysis is part of a larger study beyond the focus of this paper.

The peptide library was generated with the help of BISC Global, Ghent, Belgium. The Uniprot resource (https://www.uniprot.org) was used to confirm the correct length and position of the proteins from dengue (DENV1, DENV2, DENV3 and DENV4), ZIKV (Asian, African) and YFV (ECA, SA, WA) viruses. Fasta files available before September 2018 per virus, lineage and protein were retrieved from the ViPR database https://www.viprbrc.org. The retrieved data represented a wide geographical coverage including countries from Southeast Asia, East Asia, America and Africa.

The variable and conserved regions were compared among the downloaded isolates after aligning the sequences using Jalview (https://www.jalview.org). Based on the conservation degree of >75% per AA of the analysed sequences and the AA properties, one or multiple unique sequences per virus, lineage and protein were obtained with optimal global coverage. Finally, a total of 284 unique consensus sequences (135 for DENV, 28 for ZIKV, 72 for YFV and 49 for CHIKV) were retrieved from this analysis to generate the peptide library.

The unique sequences were cut into pentadecapeptides (15-mer) with a consecutive overlap of 11 residues in order to create unique short peptides. The resulting 9,023 peptides covered the proteomes from DENV, ZIKV, YFV and CHIKV completely: 8,963 were unique peptides (4,444 for DENV; 1,134 for ZIKV; 1,913 for YFV; 1,472 for CHIKV) and 60 were non-unique peptides (present in more than one flavivirus). Additionally, 49 pan-flavivirus reactive peptides identified previously using another microarray (unpublished) were also included in the peptide library. Peptides were mapped to the Uniprot reference strains for DENV (P33478), ZIKV (A0A142I5B9), YFV (P03314) and CHIKV (Non-Structural Proteins: Q5WQY5, and Structural Proteins: A0A1I7PCZ2).

The peptide library covered more than 70% of the global diversity of available sequences from DENV, ZIKV, YFV and CHIKV sequence identities available in the ViPR database. The density of peptides in the library varied across the proteome (**Supplementary Figure S1**). For DENV, the array included on average 7 peptide variants per each location with a maximum of 10 variants for the most variable regions in the proteome.

### Microarray Peptide Synthesis, Immunolabeling and Pre-Processing

The peptide microarray synthesis, immunolabeling assay and image processing were done in collaboration with Schafer-N (Copenhagen, Denmark) and the AIT Austrian Institute of Technology GmbH (Vienna, Austria). A random position was assigned to each peptide on the array slide to minimize the impact of locational bias. The peptide set (interest and control peptides) was deposited in twelve identical sub-arrays per slide, enabling the analysis of 12 samples simultaneously. The 48 serum samples included in the analysis were chemically inactivated with Triton X-100 0.1% before performing the immunolabeling. The non-specific binding sites in the slides were blocked with 0.1% Bovine Serum Albumin in PBST (blocking buffer) for 1 h at room temperature. After removing the blocking buffer, the serum, diluted 1:100 in blocking buffer, was applied and incubated on a rotator for 1 h at room temperature. Slides were then washed with blocking buffer and incubated for 1 h at room temperature with polyclonal Alexa Fluor 647 conjugated goat anti-human IgM (1 µg/ml) and Cy3 conjugated goat anti-human IgG (1 µg/ml). The slides were then washed with blocking buffer, spin dried and scanned. The images were analyzed using the PepArray program (Schafer-N). This process included the subtraction of the local background surrounding the peptide fields. The background corrected intensities were converted into arbitrary units of fluorescence intensity in 8-bit format. The data was finally transformed to 16bit for further analysis.

### Data Processing

The array data (MFI values) was quantile normalized using the add-in tool from JMP^®^ Pro, Version 14.0. SAS Institute Inc., Cary, NC, 1989-2019. Results from each peptide were categorized to their respective protein, lineage and virus using a custom-designed R script. Another custom designed R script was also created for the calculation of the threshold value using the expectation–maximization (E-M) algorithm, in which a bimodal curve is created per sample based on the spread of the noise- and signal distribution. The mean of the noise distribution +2SD (Standard Deviation; based on a p < 0.01 in order to have <1% chance to false positives) was defined as the cutoff value for a positive signal. Then, the calculated threshold was subtracted from the MFI to every peptide signal in each sample. Each value below the threshold was considered negative and changed to “0”.

### Mapping the Magnitude, Depth and Breadth of the IgG and IgM Antibody Response to Linear Peptides

First, the number of hits, i.e., the peptides above the cut-off (IgG or IgM reactive peptides) at each time point in every sample were normalized to the number of peptides included in the microarray per virus and per protein. Heatmaps were created based on the calculated percentages.

Second, the humoral responses against DENV, ZIKV and YFV were visualized independently by plotting the magnitude of IgG and IgM Ab binding, measured in arbitrary fluorescence intensities by peptide location (starting amino acid position). The R packages pepStat, pepDat, ggplot2, Pviz and Gviz were used to plot the magnitude of the Ab response in terms of fluorescent intensities to individual peptides, categorized by protein and amino acid start position as aligned to the proteome of the Uniprot reference strains for DENV (P33478), ZIKV (A0A142I5B9) and YFV (P03314). Individual plots per time point and per virus were generated.

Third, antibody target regions (ATR) were calculated to represent the breadth and depth of the humoral response. We first identified peptides above the cut-off (IgG or IgM reactive peptides) in at least 5 or more patients for the endemic group and 2 or 3 in the traveler group, for further analysis. If two reactive peptides shared an identical sequence of 5 or more contiguous AAs, peptides were considered as a single positive binding site and called an ATR. This approach is based on established methods to analyze Ab breadth as described in Stephenson et al. (66). Then, the breadth of the Ab response was defined as the number of non-overlapping ATRs across the polyprotein in each virus. Because ATRs are calculated based on reactive overlapping peptides, the breadth of the response should be interpreted considering not only the number of ATRs but also their width (length in terms of number of AAs). Special attention was given when comparing the breadth of Ab responses between viruses. The depth of the Ab response was evaluated based on the number of unique sequence variations per ATR (classified by serotype for DENV and lineage for DENV, ZIKV, YFV) recognized by each sample among the groups. The selected ATRs were plotted using a custom-designed R script.

Fourth, the fraction of patients (> 5 patients for the endemic group and 2 or 3 in the traveler group) that recognized a 15-mer peptide at each position across the virus polyprotein, were compared for IgG and IgM responses. Plots for each protein and for each time point were created using JMP^®^, Version 15. SAS Institute Inc., Cary, NC, 1989-2019. software.

Finally, a longitudinal humoral response analysis based on the Ab fold change in the early-, mid- and late-convalescent samples, in respect to the acute sample, was done per each patient included in the study. When the fluorescence intensity was “0” in the acute sample, the FI value from the convalescent samples was used for plotting. Plots for each patient were created using JMP^®^, Version 15. SAS Institute Inc., Cary, NC, 1989-2019. software.

### Identification of Flavivirus Cross-Reactive and DENV-Specific Peptides

We created individual alignments for each flavivirus protein using the sequences retrieved for the microarray design. The conservation rates (percentage identity) between the amino acid positions were represented in bar plots using a sliding window size of 15 amino acid positions. Conservation plots were created using Geneious Prime 2020.2.4. Based on these alignments, the DENV, ZIKV, YFV peptides were aligned onto the DENV polyprotein (Uniprot: P33478). Reactive peptides from DENV1-4, ZIKV and YFV targeted by IgG and IgM Abs from at least five out of the 10 endemic patients and two or three traveler patients were plotted together at each time-point. Individual graphs per each protein, mapping the aligned flavivirus peptides were created using a custom-designed R script.

Based on this analysis, PanFlavi (overlapping DENV and ZIKV and/or YFV peptides) and DENV-specific (DENV peptides showing non-overlapping reactivity with ZIKV or YFV peptides) regions, that were reactive at any time-point of the follow-up, were schematized.

Finally, individual heatmaps were created for the more representative selected peptides using JMP^®^, Version 15. SAS Institute Inc., Cary, NC, 1989-2019. software.

## Results

### Antibodies From DENV-Infected Individuals Target Epitopes in Linear Peptides From DENV, ZIKV and YFV

**Figure 1:**
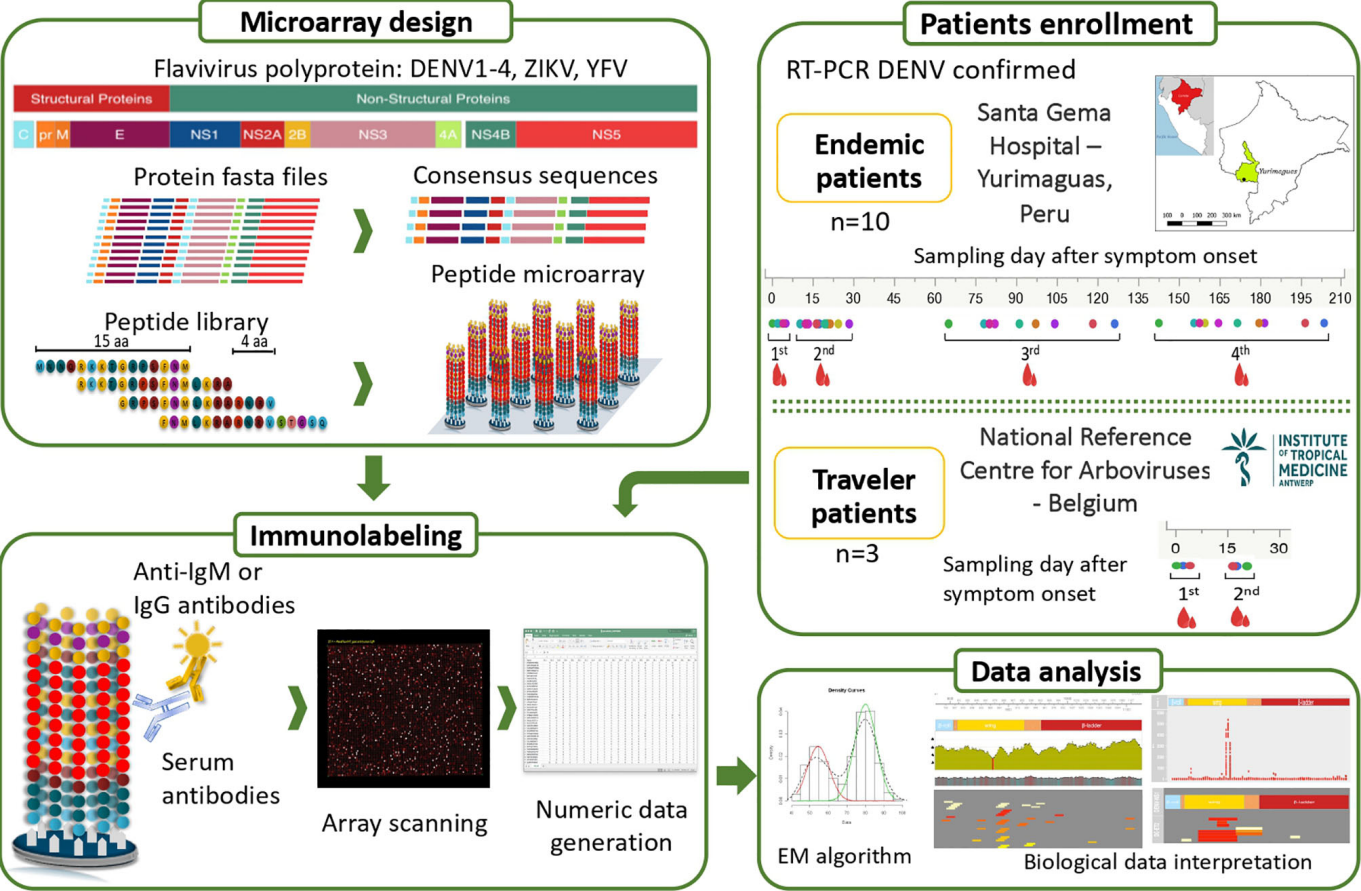
Overview of the study design. Two groups of febrile patients: (i) endemic individuals coming from the Peruvian Amazon and (ii) Belgian returning travelers were enrolled. DENV infection was confirmed by RT-PCR. Four blood samples were collected over six months after symptom onset in the endemic group and two samples within a month after onset of symptoms in the traveler group. The bioinformatic analysis to design the peptide microarray included downloading fasta files from the ViPR database https://www.viprbrc.org, representing a wide geographical coverage of DENV, ZIKV, YFV and CHIKV. Based on the conservation degree of the analysed sequences and the AA properties, one or multiple sequences per virus, lineage and protein were obtained with optimal global coverage, which were finally used to generate the peptide library. The 284 unique sequences were broken up into 9,023 peptides, each of 15 amino acids (aa) length tiling every 4 AA across the DENV, ZIKV, YFV and CHIKV proteomes. The synthetized peptide library was mixed with sera containing antibodies that bind to their cognate epitope on the peptide. Bound antibodies were stained with either anti-IgG– or anti-IgM–secondary antibodies. Lastly, the peptide microarrays were scanned and the image files were converted to numeric data. Cut-offs were calculated per sample using the expectation maximization (EM) algorithm and the data was used for further analysis.

To measure the humoral response, we compared IgG and IgM reactivity profiles in our custom-designed microarray from a cohort of DENV2-infected individuals from an endemic area in Peru (representative for arbovirus-experienced individuals) to those from infected individuals with DENV after visiting endemic areas (Belgian travelers representative for a primary infection) (**Figure 1**). This comparison facilitated the identification of peptides that elicited specific and CR-Abs as well as the mapping of conserved immunodominant regions among the flaviviruses recognized by endemic and traveler individuals after a symptomatic DENV infection.

**Figure 2:**
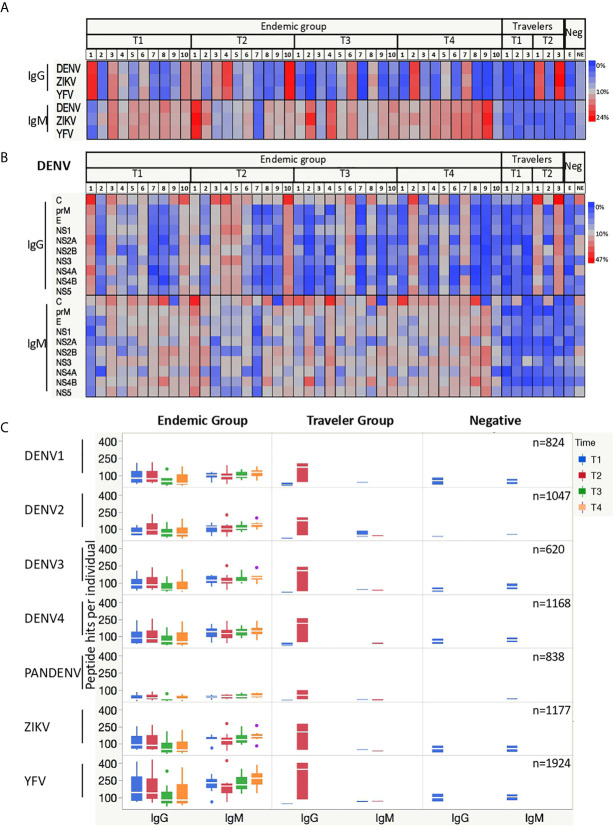
Antibodies from DENV-infected individuals target epitopes in linear peptides from DENV, ZIKV and YFV. **(A)** Heatmaps depicting the relative number of peptides targeted by IgG and IgM Abs present in the sera from endemic (n=10) and travelers (n=3) at the time samples were collected: T1 (acute), T2 (early convalescent), T3 (mid convalescent) and T4 (late convalescent). Each column represents one sample. Samples from the negative subjects are the two last columns (E: endemic, NE: non-endemic). The color intensity indicates the relative number of 15-mer peptides from the indicated arbovirus against which IgG and IgM antibodies are reactive. **(B)** Heatmaps (as in **A**) of the relative number of peptides from each DENV protein targeted by IgG and IgM Abs. Each column represents a sample, and each row represents a DENV protein. **(C)** Box plots illustrating the number of peptide hits targeted by IgG and IgM Abs present in the sera from DENV patients and negative samples. The box indicates the interquartile range, with the line at the median. The time points are color coded as follows: acute (blue box), early convalescent (red box), mid convalescent (green box) and late convalescent (orange box) samples. The boxes for the negatives are in blue. The numbers in the right corner in each row indicate the total number of peptides included in the microarray for each virus.

Interestingly, similar percentages of DENV, ZIKV and YFV peptides were targeted by Abs from DENV infected subjects at individual level (**Figure 2A**). In the endemic group, the relative number of IgG-targeted peptides together with the onset of the Ab responses differed markedly between individuals, whereas the percentage of peptides targeted by IgM Abs was more homogeneous between subjects and constant over time. For the travelers, the percentages of IgG-targeted peptides increased from acute to convalescent samples, while for IgM the reactive peptides remained constant with low percentages in both time-points.

When analyzing the percentages of reactive peptides from each protein of the DENV polyprotein targeted by IgG Abs, the capsid (C), envelope (E), NS1, NS3 and NS5 proteins showed the highest values. This reactivity was consistent between individuals for IgG, whereas for IgM only the C protein was homogeneously and constantly targeted, while the response against the other DENV proteins differed per individual (**Figure 2B**). Given that all endemic patients were infected with DENV2, we would expect an increased reactivity against DENV2 peptides, however when we stratified the response into the four DENV serotypes plus those peptides with shared sequences by more than one serotype (PanDENV), no skewed response was observed towards the DENV2 serotype. When the Ab responses against ZIKV and YFV peptides were also stratified into the ten proteins, a similar preference towards the most immunogenic proteins was observed (**Supplementary Figure S2**).

We then plotted the absolute number of DENV peptides targeted by IgG and IgM Abs. We observed that in the endemic group, the number of IgG-targeted peptides increases from the acute to the early-convalescent samples and then declines over time, while for IgM the number remains quite constant across time (**Figure 2C**). Also noticeably were the high number of ZIKV and YFV peptides targeted by IgG Abs early in the acute phase of the disease by the endemic group, in contrast to the low number observed in the travelers.

### Humoral Responses to Linear Epitopes Covering the DENV, ZIKV and YFV Proteome

**Figure 3:**
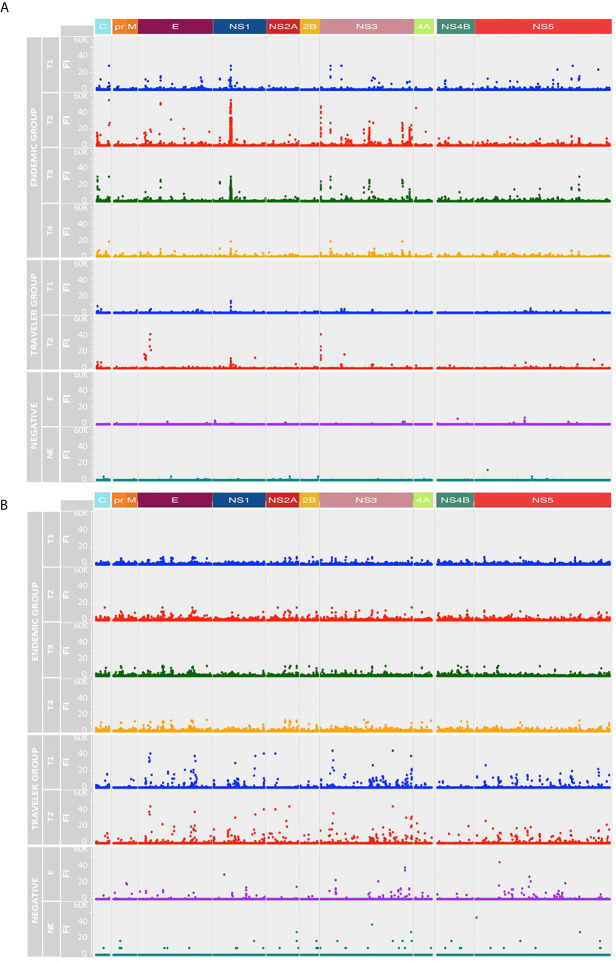
Longitudinal humoral responses to linear epitopes covering the DENV proteome. The plots show the level of IgG **(A)** and IgM **(B)** Ab reactivity measured in fluorescence units relative to proteomic coordinates for DENV (Uniprot ref: P33478). Structural: Capsid (C), Membrane (pr, M) and Envelope (E), and non-structural proteins: NS1, NS2A, NS2B, NS3, NS4A, NS4B and NS5 are color represented; each dot represents the Ab binding to a single peptide in the microarray. In both A and B plots, the endemic group is at the top (four-time points), the traveler group at the center (two-time points) and the negatives at the bottom (one-time point). T1: acute (blue dots), T2: early convalescent (red dots), T3: mid convalescent (green dots) and T4: late convalescent (orange dots) samples. Responses from the endemic (E) and non-endemic (NE) negatives are represented in purple and cyan dots, respectively.

The IgG and IgM binding pattern against each peptide spanning the DENV proteome is shown in **Figures 3A, B**, respectively. What stands out in this figure is the different reactivity pattern seen between IgG and IgM Abs. While the IgG Ab responses were directed towards discrete regions preferentially located at the C, E, NS1, NS3 and NS5 proteins, the IgM responses were characterized by being scattered across the DENV polyprotein, making it difficult to identify regions preferentially targeted by these Abs.

IgG Abs from the endemic and traveler groups targeted regions directed towards the N- terminal of C, the DI/DII hinge region of E, the wing domain of NS1, the N-and C-terminal protease domain and DIII of NS3, and the finger and palm domains of NS5. The highest Ab signal intensities were directed towards peptides located in the wing domain of NS1. Differences between both groups were seen in the high IgG levels directed against regions located in the C (C-terminal), the E (DI and TM) and the NS3 (protease domain, the DI and DIII) in the sera from endemic samples, compared to low or absent reactivity against the same regions in the travelers. Through this visual analysis, we were not able to identify regions that are exclusively targeted by Abs in sera from primary infections.

As expected, the IgG and IgM reactivities from the traveler and endemic groups showed patterns consistent with the described humoral responses for primary and secondary infections, respectively. On one side, the traveler group showed a rapid rise of IgM Abs AOS and a gradual increase of IgG levels, while the humoral response in the endemic patients was characterized for presenting low and steady levels of IgM and a rapid increase of the IgG response with high Ab levels.

In the endemic group the peptide reactivities changed over time and differed between IgG and IgM Abs. By visual analysis, the IgG response followed a rise and fall pattern, where fluorescence intensity values peaked in the early convalescent samples, waned towards the mid convalescent sample and reached the lowest levels by month six AOS (**Figure 3A**). By contrast, the IgM response remained steady throughout the time interval studied (**Figure 3B**). Sera from the negative controls showed reactivity against some peptides in the DENV proteome, although the recognized peptides were fewer in number and lower in magnitude compared to the responses from DENV-infected individuals.

**Figure 4:**
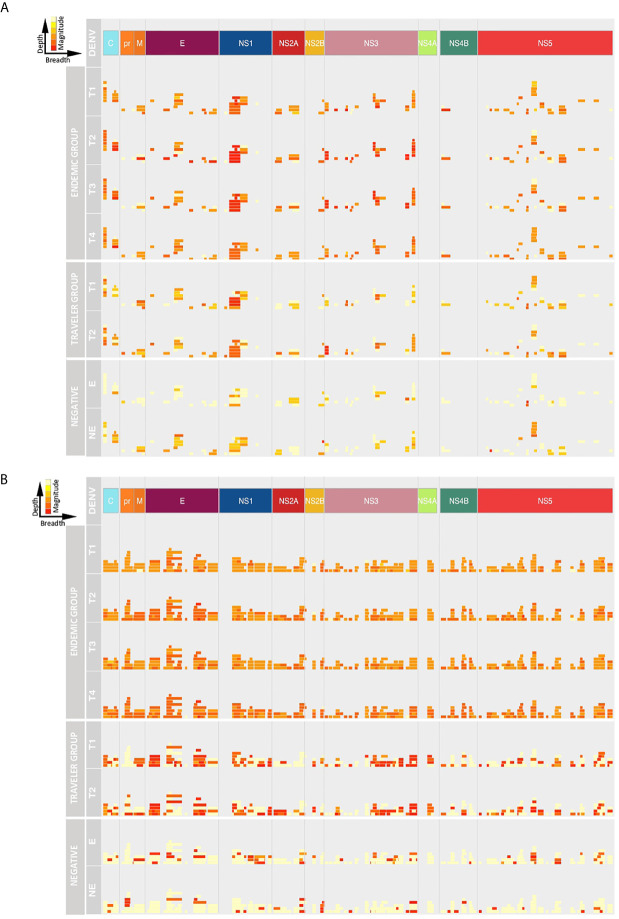
Depth and breadth of the humoral response against DENV peptides. The plots show the Ab target regions (ATRs) for IgG **(A)** and IgM **(B)** aligned to the proteomic coordinates for DENV (Uniprot ref: P33478). Structural: Capsid (C), Membrane (pr, M) and Envelope (E), and non-structural proteins: NS1, NS2A, NS2B, NS3, NS4A, NS4B and NS5 are color represented. Each bar in the plot represents an ATR. The depth (different epitope variants recognized by the sera) of the Ab response is read vertically, the breadth (binding sites across the polyprotein) of the Ab response is read horizontally and the color intensity of each ATR indicates the magnitude of the Ab response, in terms of arbitrary fluorescence intensity units after Log2 transformation (ranging from 0 to 16). T1: acute, T2: early convalescent, T3: mid convalescent and T4: late convalescent samples. E: endemic and NE: non-endemic negative samples.

Next, we analyzed the breadth and depth of the Ab responses towards the DENV proteome over time based on the calculated ATRs. In this study, the depth of the IgG and IgM responses in the travelers was lower compared to the endemic group, however, the IgG depth increased in the convalescent samples in both groups (**Figure 4A**), while the IgM depth remained stable (**Figure 4B**). The IgG depth was greater in specific regions of the polyprotein mainly in the C, E, NS1, NS3 and NS5 proteins, meaning that the IgG Abs recognized a broader sequence diversity in these immunodominant regions. For the negative controls, despite the low Ab levels observed in the intensity maps, a strikingly similar pattern in depth and breadth with respect to the DENV-infected individuals was observed. This may be explained by (i) a general background of epitopes probably targeted by natural Abs, (ii) the use of a Log2 scale for the fluorescence intensity values in the representation of the color intensities of the ATRs in **Figure 3** and that (iii) calculation of the ATRs is based on a unique result: one serum sample in the endemic control and a pool of sixteen sera in the non-endemic control.

Despite the small number of variants recognized by IgM Abs in the travelers, the magnitude of the IgM response towards these ATRs was higher compared to the response elicited by endemic patients that showed a IgM response higher in depth but lower in magnitude.

In the endemic and traveler groups, the breadth of the IgG Ab response was lower in terms of ATR numbers across the polyprotein and also these ATRs were characterized for being shorter in length compared to the IgM response. This is in accordance with the findings mentioned above that the IgG Abs were directed towards discrete regions of the polyprotein, while IgM Abs targeted regions scattered across the entire polyprotein. The Ab breadth differs between groups over time, for the endemic group the breadth remained constant during the follow-up period and in the traveler group the breadth was higher in the convalescent samples compared to the acute time points.

Taken together, these results show that for the endemic group, the IgG response increased from the acute to the early convalescent time point in terms of depth and magnitude, while the breadth remained stable and, in the travelers, these three features increased over time. For the IgM response, the depth, magnitude and breadth were similar in both groups, they remained stable over the studied time points in terms of the magnitude and breadth, but the depth increased over time.

Overall, the IgG and IgM Ab responses against peptides from ZIKV and YFV in the endemic and traveler groups followed a similar pattern as for DENV, both for the targeted antigens and for the temporal evolution of Ab responses (**Supplementary Figures S3**). However, the major differences in the response against these viruses were found in the magnitude and breadth of the response. Thus, in the endemic group, the magnitude together with the breadth of Ab responses against ZIKV and YFV was lower compared to the responses seen against DENV (**Figure S4**).

To determine if the responses against ZIKV or YFV in the endemic group was higher in those patients that have NAbs against these viruses, we divided the samples into those with presence or absence of NAbs and plotted heatmaps based on the IgG and IgM signal intensities of the ATRs. No difference was observed between the split groups (**Supplementary Figure S14**).

For the travelers, despite the fact that the number of ZIKV and YFV peptides targeted by IgG Abs were similar to the DENV peptides, the fluorescence intensity values were lower (**Supplementary Figures S3A**). This was not the case for the IgM response, of which the levels were remarkably high in this group, especially against peptides located in the E, NS1, NS3 and NS5 proteins (**Supplementary Figures S3B**) with similar fluorescence intensity values as the ones detected against DENV peptides, but with fewer ATRs (**Supplementary Figures S4**). Since individuals from the traveler group experienced a primary infection, it is likely that this response is a measure of CR-Abs elicited towards conserved flavivirus immunodominant epitopes.

### Identification of the Most Frequently Targeted Regions Recognized by IgG and IgM Abs

**Figure 5:**
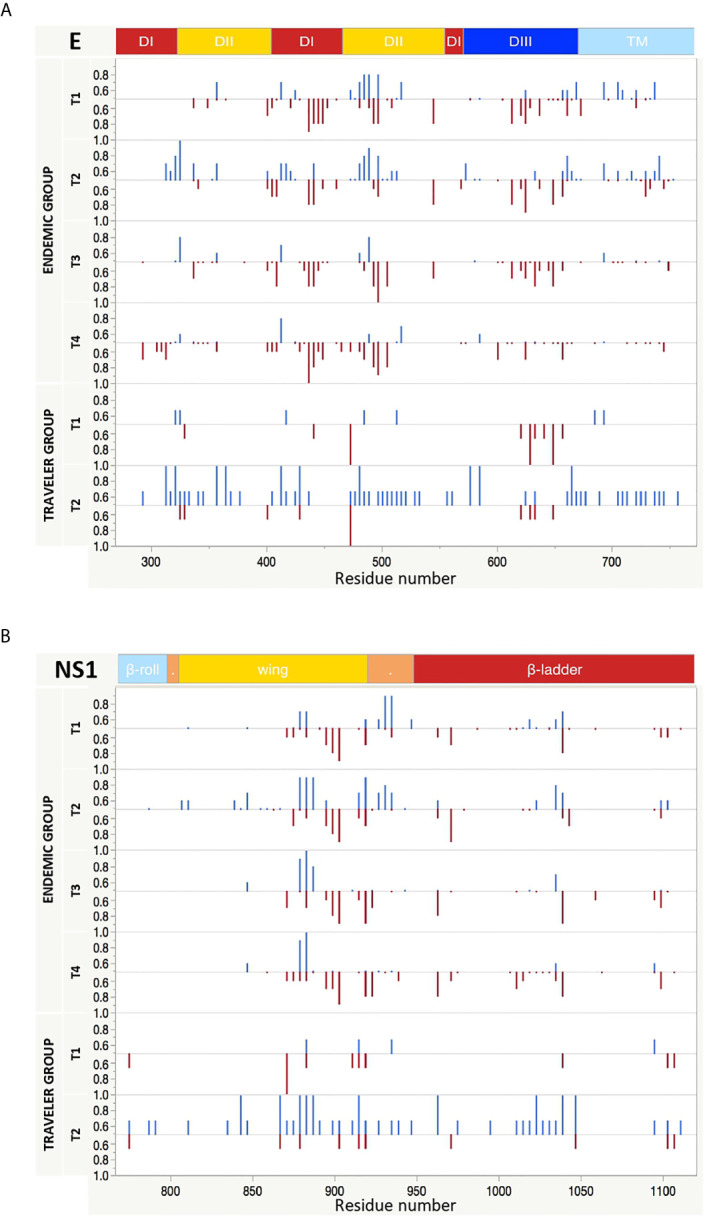
Longitudinal IgG and IgM reactivity against E and NS1 proteins from DENV. The y axis indicates the fraction of endemic (n = 10) or traveler (n=3) patients reacting against peptides from E **(A)** and NS1 **(B)** proteins with either IgG (top, blue bars) or IgM (bottom, red bars) antibodies at the time points samples were collected. T1: acute, T2: early convalescent, T3: mid convalescent and T4: late convalescent samples. The x axis indicates the proteomic coordinates (amino acid start position) according to DENV Uniprot ref: P33478. The protein domains for E and NS1 are color represented at the top of each plot.

Next, we compared the fraction of patients that patients that have IgG and IgM antibodies against immunodominant regions. Overall for DENV, few regions were concurrently targeted by both antibody isotypes. These regions could be identified in domain II of the envelope (EDII), the wing domain of NS1, the protease domain of NS3 and the finger domain of NS5. There were also regions preferentially targeted by one specific isotype, such as the regions located in the DI from E, the C-terminal of the NS1 wing domain, the domain I of NS3 and the MTase and the thumb domains of NS5, which were more frequently targeted by IgM Abs; or the regions at the DI/DII hinge of the E, the greasy finger of the wing domain of NS1 and the C-terminal of the finger domain of NS5 that were mainly targeted by IgG Abs (**Figure 5** and **Supplementary Figures S5**). We also observed that the levels of Ab reactivity did not always correspond with the frequency of patients responding against a specific region. This is the case of NS2A and the C-terminal part of NS5 that showed low IgG levels (**Figure 3**), but were frequently recognized by the DENV-infected individuals (**Supplementary Figures S5**).

When we compared the response against DENV peptides with the response against ZIKV and YFV, we noticed a low frequency of patients responding against ZIKV and YFV peptides (**Supplementary Figure S6**). In general, the Ab reactivity in the endemic and traveler groups against non-DENV flaviviruses was lower in magnitude and in numbers of reactive individuals. However, there were also regions such as the hinge of EDII-ZIKV, C-terminal of prM-ZIKV, NS4B-ZIKV, C-terminal of NS2B-YFV and NS5-YFV proteins with low IgG levels but with a high fraction of patients reacting against them.

### Longitudinal Analysis of the Ab Response

**Figure 6:**
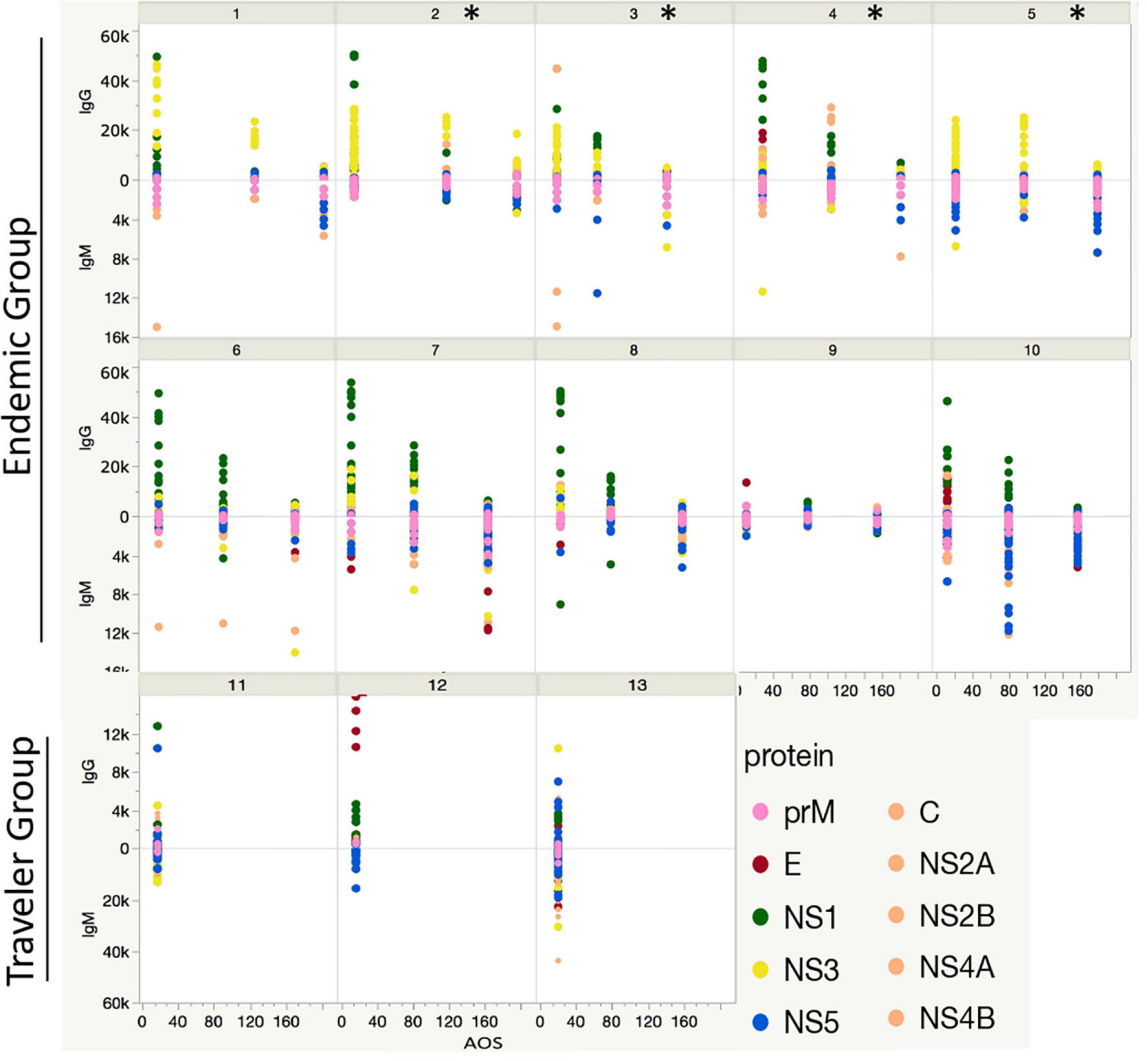
Longitudinal humoral response to DENV proteins. Each box represents the individual response of each patient through time in the endemic (from 1 to 10) and traveler (11 - 13) groups. The y axis indicates the Ab fold change per patient at the early-, mid- and late-convalescent samples, relative to the acute sample, colored by protein target. The x axis indicates the days when the convalescent samples were collected. The * symbol refers to patients presenting severe symptoms of dengue and needed hospitalization.

A longitudinal analysis of the IgG and IgM responses per each patient was performed by calculating the fold change of the fluorescence intensity levels in the convalescent samples (early, mid and late) in respect to their acute samples per each peptide. This analysis revealed two patterns of IgG seroconversion in patients from the endemic group (**Figure 6**). One pattern, for patients 1, 2, 3 and 5 was characterized for a rapid increase of IgG levels towards NS3 peptides from the acute to the early-convalescent sample that later declined over time. Interestingly, subjects 2, 3, and 5 developed severe symptoms of dengue and needed hospitalization (**Table 1**). The second pattern, for patients 4, 6, 7, 8 and 10 was mostly characterized by high fold change values towards peptides from NS1 protein in the early convalescent sample (**Figure 6**). The fold change values for the E and NS5 peptides were lower and sustained over time. For the IgM response, the seroconversion patterns were slightly divergent between patients, but overall showed higher scores for peptides from the prM and the NS5 proteins. The fold change values for prM peptides remained constant over time, while for NS5 peptides differed markedly between individuals (**Figure 6**).

For the travelers the considerable divergence in the fold change IgG and IgM values towards peptides between individuals made it difficult to identify a preferential Ab pattern against a particular protein.

We also performed the same longitudinal analysis for ZIKV and YFV peptides. The seroconversion patterns for IgG and IgM Abs were similar as those described for DENV, with the main difference that the fold change values were significantly lower for ZIKV and YFV (**Supplementary Figures S7**). For the endemic group, contrasting with the high IgG seroconversion values against NS3 peptides from DENV and ZIKV, the seroconversion values for YFV-NS3 were negligible. The seroconversion patterns in the traveler group were different between individuals and also between ZIKV and YFV and at the same time, they differed in pattern compared to DENV.

### Identification of DENV Specific and Flavivirus Broadly Immunoreactive Epitopes

Next, we mapped the flavivirus peptides from DENV, ZIKV and YFV that were recognized by >50% of DENV-infected patients onto the individual coordinates of the structural and non-structural DENV proteins (DENV1, Uniprot: P33478) at the different time-points of the follow-up.

The simultaneous mapping of flavivirus peptides targeted by the traveler individuals revealed that together with a high number of DENV peptides recognized by IgG Abs in the early convalescence samples, an extensive number of ZIKV and YFV peptides were also targeted; while fewer peptides were targeted by IgM Abs, most of them corresponding to DENV peptides, with small reactivity towards ZIKV and YFV peptides. For the endemic group, the opposite was observed, a larger number of co-localized DENV, ZIKV and YFV peptides were targeted by IgM Abs, while for IgG the reactivity was directed towards fewer overlapping regions for the three flaviviruses (**Supplementary Figures S8**–**S10**). Abs from the traveler group that simultaneously targeted DENV and ZIKV or YFV co-localized peptides are likely to be CR-Abs, while those Abs from either the endemic and the traveler group that mainly targeted DENV peptides, with low or absent reactivity towards ZIKV or YFV peptides are most likely TS-Abs. Then, these immunodominant regions likely to raise CR-Abs are called here PanFlavi and those able to elicit TS-Abs called DENV-specific.

**Figure 7:**
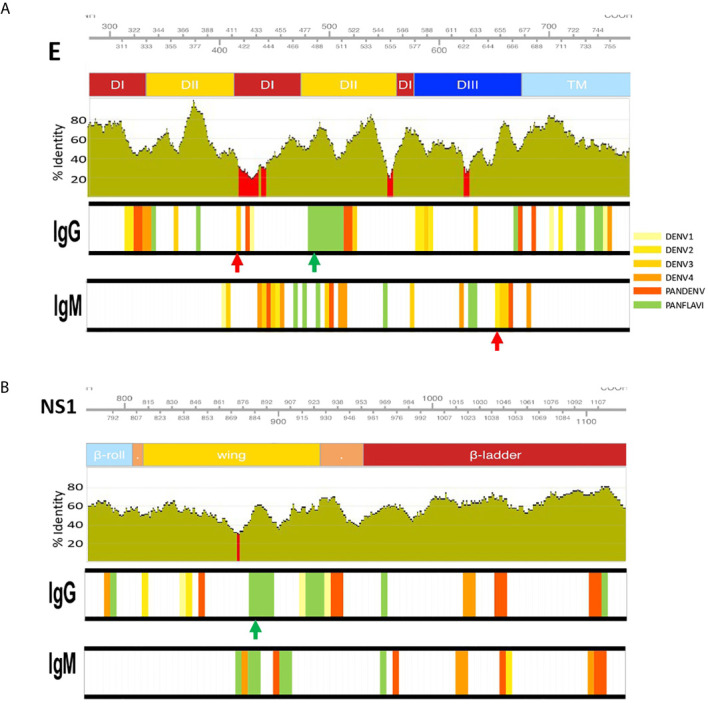
Identification of DENV specific and flavivirus broadly immunoreactive epitopes. IgM and IgG targeted epitopes at the E **(A)** and NS1 **(B)** proteins from DENV, ZIKV and YFV viruses, recognized by DENV patients were filtered out and mapped to the proteomic coordinates of DENV (Uniprot ref: P33478). Colored bars from light-yellow to orange represent immunodominant regions belonging to one DENV serotype. Red bars represent immunodominant regions belonging to more than one serotype (here called PanDENV). Green bars represent immunodominant regions belonging to DENV and ZIKV or/and YFV here called PanFlavi. E and NS1 sequences from DENV1-4, ZIKV and YFV were aligned and the conservation rate (percentage identity) between the amino acid positions is represented as green bar plots using a sliding window size of 15 amino acid positions. Red regions correspond to percentage identities below 30%. Red and green arrows are DENV-specific and PanFlavi peptides, respectively, and for which the Ab responses are further detailed in **Supplementary Figure S13**.

In order to simplify the schematization of the PanFlavi and DENV-specific regions, the peptides that were positive at any time point of the follow up were filtered out and mapped based on their start amino acid position onto the E and NS1 (**Figure 7**) and C, prM, NS2A, NS2B, NS3, NS4A, NS4B and NS5 (**Supplementary Figures S11**, **S12**) sequences. The identified PanFlavi and DENV-specific peptides, targeted by IgG and IgM Abs are listed in **Tables 2**, **3**, respectively.

**Table 2:** Most reactive IgG peptides selected by the high-density microarray.

	Type	Protein	Protein position		Amino acid length
			start	end	
IgG	PanFlavi	C	1	23	23
		C	61	100	40
		prM	117	155	39
		E	197	239	43
		NS1	105	131	27
		NS2A	85	99	15
		NS2B	109	130	22
		NS3	1	31	31
		NS3	321	335	15
		NS3	337	351	15
		NS5	369	391	23
		NS5	465	491	27
	DENV-Specific	prM	29	47	19
		E	33	67	35
		E	133	147	15
		E	293	319	27
		NS1	61	87	27
		NS2A	141	179	39
		NS2B	89	103	15
		NS3	477	491	15
		NS3	585	603	19
		NS4A	29	47	19
		NS4B	204	218	15
		NS5	321	339	19

**Table 3:** Most reactive IgM peptides selected by the high-density microarray.

	Type	Protein	Protein position		Amino acid length
			start	end	
IgM	PanFlavi	C	61	100	40
		prM	33	67	35
		prM	97	163	67
		E	181	219	39
		E	337	359	23
		NS1	93	143	51
		NS3	57	135	79
		NS3	309	355	47
		NS4B	5	43	39
		NS5	89	123	35
		NS5	177	223	47
		NS5	269	299	31
		NS5	509	547	39
	DENV-specific	E	153	187	35
		E	213	243	31
		E	365	391	27
		NS1	265	283	19
		NS1	321	343	23
		NS2B	109	130	22
		NS3	201	227	27
		NS3	465	491	27
		NS3	593	611	19
		NS4A	89	107	19
		NS4B	73	107	35
		NS4B	193	218	26
		NS5	469	483	15
		NS5	869	883	15
		NS5	881	899	19

For the E protein, this analysis revealed five immunodominant regions recognized by IgG Abs (**Figure 7A**). One located at EDII_474-511_ of the DENV proteome is likely to be PanFlavi, since the same regions in E-ZIKV and E-YFV were recognized not only by endemic patients but also by travelers. Another three regions are likely to be DENV-specific. These regions located in EDI/II_311-338_, EDI/II_411-443_ and EDIII_622-637_ were frequently recognized by Abs in the endemic and traveler groups but their homologous regions in ZIKV and YFV showed minimal or no reactivity. The last immunodominant region was identified at EDII_356-371_ and is likely to be DENV-specific with the difference that it was targeted preferentially by the endemic group but not by the travelers. Coincidentally, the PanFlavi regions correspond with regions that are highly conserved among DENV serotypes, ZIKV and YFV, which could explain the high reactivity among them. In contrast, the DENV-specific epitope sequences were located in regions with low identity percentages among these viruses.

For E regions targeted by IgM Abs, we identified five immunodominant regions, the PanFlavi epitopes were located in EDI/DII_455-499_ and EDIII_625-639_, while the DENV-specific sequences were identified in EDI_434-465_, EDII_500-522_ and EDIII_649-675_ (**Figure 7A**). The highly conserved fusion loop (FL) was recognized by IgG Abs in three out of ten endemic patients and in two out of three travelers, and by IgM Abs in only one out of the ten endemic patients (**Supplementary Figure S11**).

For NS1, five immunodominant regions were identified to be most frequently targeted by IgG Abs (**Figure 7B**). One region, highly conserved among the flaviviruses was located in the wing domain of NS1_877-906_ and it was targeted by IgG Abs from individuals of the endemic and traveler group. These Abs are likely to be cross-reactive, since not only the endemic but also the traveler individuals recognized the same region in the ZIKV and YFV proteome. Another three regions, one spanning the wing domain NS1_838-861_ and the other two in the ß-ladder at positions NS1_1033-1053_ and NS1 _1093-1114_ were also targeted by IgG Abs from endemic and traveler individuals, however sera from the traveler group recognized not only DENV peptides, but also ZIKV and YFV peptides located in the same regions, meaning that these were PanFlavi regions for the travelers but DENV-specific for the endemic group (**Supplementary Figure S11**).

A PanFlavi region in the NS1 protein spanning the wing domain from residues 859-915 was frequently recognized by IgM Abs from endemic samples. Three other regions in NS1 were likely to be DENV specific, since IgM Abs from the endemic and the traveler individuals recognized mainly DENV peptides in this region, with little or no reactivity towards ZIKV or YFV peptides (**Figure 7B**).

The more representative PanFlavi and DENV-specific peptides targeted by IgM and IgG Abs that could represent good serological markers are shown in **Supplementary Figure S13**. Notably, the DENV-specific peptides located at E_654-668_, NS4B_2396-2410_, E_413-427_, NS2B_1433-1447_ and NS4B_2312-2326_, belong to a unique DENV serotype, while regions located at NS4B_2324-2338_ and NS3_2059-2073_ belong to multiple DENV serotypes.

## Discussion

The negative impact of the increasing cocirculation of different arboviruses in endemic areas on the reliability of current serological assays highlights the necessity for the discovery of better serological markers that can solve the cross-reactivity problem. These improvements may be of benefit to the clinical management of arboviral suspected cases given the risk of severe clinical manifestations in secondary infections, but will also help to better define DENV serostatus required for vaccination strategies with Dengvaxia. In this work, we conducted for the first time a high-throughput longitudinal scanning of the diversity of IgM and IgG Abs using a proteome-wide microarray containing overlapping linear peptides from DENV, ZIKV, YFV and CHIKV in sera from individuals infected with DENV.

A fundamental aspect of this work is the comparison of the longitudinal IgM and IgG Ab profiles to the entire viral proteome in individuals from endemic areas, which most likely experienced previous arboviral infections, in contrast to the Ab response from individuals that experienced a first DENV infection after visiting an endemic area. Our results demonstrated that IgM and IgG responses towards DENV linear peptides in primary infected travelers and in individuals living in endemic areas were in agreement with the dynamics of primary and secondary infections, respectively.

We found that the IgG response differed in magnitude across time and across the proteome, with the strongest responses in the early convalescent phase and with the most immunodominant peptides located in proteins C, prM, E and NS1. This is in accordance with previous work showing that these proteins are the main targets of the Ab responses (41). Although NS3 and NS5 proteins are known to contain T cell epitopes mainly targeted by CD8+ T cells (42, 43), our results showed that these proteins also have epitopes targeted by IgG antibodies.

The observed low levels of IgM Abs in the endemic samples compared to the response in the travelers has been previously reported, indicating that secondary infections with homologous viruses are characterized by the production of lower levels of IgM compared to the response in primary infections (44, 45). This has been linked to the “original antigenic sin” phenomenon, stating that prior exposure induces an ineffective response or even absence of response to a related antigen upon re-exposure. Although IgM Ab response in primary viral infections is usually transient in nature (23, 45), other studies have reported the long persistence of IgM in DENV-primary infected individuals (46). Here, in agreement with these studies, we found that IgM Abs persisted at sustained levels and even 6-months AOS in the endemic group. Unfortunately, we were unable to follow the evolution of the IgM response in the traveler group because we only had an acute and early convalescent sample for this group. Taken together, these studies imply that using IgM could be problematic in providing a differential and accurate diagnosis because it could render: (i) a false-positive result due to the reaction to related viruses by CR-Abs (47–49), (ii) a false positive result of a current infection as a consequence of TS- or CR- long-lived IgM Abs (50), or (iii) a false negative result due to the absence of IgM in secondary flavivirus infections (46).

The high IgG titers appearing very early AOS in the endemic group, compared with the magnitude of the response in the traveler group is consistent with an anamnestic humoral response reported previously as the result of a “boost” in Ab titers when homologous reinfection or a low-level heterotypic infection takes place (1, 25, 51). With yearly DENV outbreaks occurring during the rainy seasons in Peru, it is very likely that the high IgG levels detected early AOS are the result of re-exposure with a homotypic virus in the past. Secondary flavivirus exposures are common in endemic areas where there is cocirculation of multiple dengue serotypes plus other flaviviruses with an increasing prevalence such as ZIKV, and programs of YFV vaccination (2), resulting in a complex multiply primed immune system. As a consequence, the observed Ab repertoire of DENV-infected individuals from an endemic area could include re-evoked homotypic or heterotypic antibodies, new DENV2-specific Abs developed against the current infection, or long-lived specific Abs from a previous exposure towards ZIKV after a natural infection or towards YFV as a consequence of vaccination.

An unexpected finding in this study was the overall more extensive recognition of ZIKV and YFV peptides by IgG Abs compared to IgM in the convalescent samples from the travelers. Most of these regions overlapped with reactive DENV peptides, with the difference that the magnitude of the response against DENV peptides was substantially higher as compared to the response against the other viruses. We identified the Abs recognizing these regions as cross-reactive which also happened to correspond with regions of high genetic and antigenic similarity between these viruses. We were able to map the immunodominant regions across the proteome that evoked CR-Abs.

Severe manifestations of dengue are more commonly seen in secondary infections (6). The pathogenesis of dengue is multifactorial with contributions from the virus and the host, and the degree to which the humoral responses may influence the balance between protective and detrimental effects of DENV-specific immune responses is still not fully understood. However, in secondary infections suboptimal levels of CR-Abs, mainly directed against structural proteins, could drive ADE, resulting in more severe pathology (21). We found that in a sub-set of endemic patients that developed severe dengue disease, the seroconversion in the early convalescent samples was mainly dominated by high IgG scores against NS3 peptides. The role of Abs against non-structural proteins other than NS1 have been poorly studied. Because NS3 is an intracellular protein, most studies have focused on identifying T-cell immunodominant regions, reporting that epitopes located in the NTPase and helicase of NS3 are the most immunodominant in the cellular response against dengue (52, 53) and that this response towards NS3 is associated with dengue hemorrhagic fever (54). However, the mechanisms leading to this exacerbation of symptoms are not fully understood. Whether Abs directed towards peptides from NS3 protein could also play a role in the pathogenesis of the disease remains unclear and could be the focus of future investigation.

The E protein together with the PrM protein elicit most of the Ab responses against the flaviviruses. In our study, IgG and IgM Abs from endemic people and travelers recognized peptides in the prM protein from DENV1-4, ZIKV and YFV. This is in agreement with previous reports indicating that Abs directed against this protein are a major component of a cross-reactive response and are characterized by being non-neutralizing and able to induce ADE in DENV in secondary infections (6, 44, 55, 56).

Epitopes covering the highly conserved fusion loop (FL) of the E protein were not frequently recognized by the DENV-patients that we studied. However, a large proportion of anti-DENV Abs appears to be cross-reactive and to target the FL. These CR-Abs are also considered to be non-neutralizing and able to induce ADE *in vivo* and *in vitro* (57, 58). The lack of reactivity against this peptide in our study can be explained by the fact that this is a conformational epitope which is buried in the immature form of the E protein and becomes accessible during the “dynamic” breathing of the E dimers at the virion surface. The native folding of this epitope might not be present in the linear peptides covered in our microarray.

The characterization of NAbs against the E protein has revealed that the most potent DENV NAbs are directed against the E-DIII in mice while in human the NAbs seem to preferentially recognize quaternary epitopes present only in virions. Although we cannot refer to the detected Abs as neutralizing, we were able to identify a DENV-specific peptide located in the EDIII_622-637_ which region was previously reported as being targeted by NAbs (59).

High levels IgG Abs were detected and they reacted against regions from DENV1-4, ZIKV and YFV which underlies the cross reactivity in various serological assays. For example, our data showed that a highly conserved region among the flaviviruses, targeted by IgG Abs from the endemic group, is located in the wing domain of the NS1 protein which is currently used as target for the differentiation between DENV and ZIKV infections.

Some of the DENV peptides that we have identified in this study, have also been reported by previous studies to be recognized by monoclonal Abs and polyclonal sera from DENV infected individuals, especially those located in the E (60, 61) and NS1 protein (32, 62–65). However, a limitation of our linear peptide array is that conformational epitopes are lacking and thus we likely missed these epitopes in our analysis.

In summary, our study provides novel insights in the proteome-wide antibody response against dengue virus and demonstrates that peptide microarrays represent a powerful tool for mapping type-specific and cross-reactive Abs following natural infection, allowing the identification of peptides that exhibit a great potential for diagnostic purposes. To overcome the limitation of the relatively low number of samples used in this study, the diagnostic performance of the identified biomarker candidates is part of an ongoing work using a bead-based multiplex peptide immunoassay with a large panel of samples originating from individuals with different flavivirus infections. Our findings present potentially valuable biomarkers for a future generation of peptide-based diagnostics that would be more specific and easier to manufacture compared to the conventional full-length recombinant protein assays.

## Data Availability Statement

The raw data supporting the conclusions of this article will be made available by the authors, without undue reservation.

## Ethics Statement

The studies involving human participants were reviewed and approved by Ethical review boards of the Peruvian University Cayetano Heredia, Lima, Peru (Protocol N° 101480); the Institute of Tropical Medicine Antwerp, Belgium (Protocol N° ITG 1304/19) and the University of Antwerp, Belgium (Protocol N° 19/42/477). Written informed consent to participate in this study was provided by the participants’ legal guardian/next of kin.

## Author Contributions

FF-A wrote the manuscript text. KK and FF-A implemented the analysis. KA, FF-A, KK, and KB conceived the study. FF-A, XM, and JM processed samples. FF-A analyzed the data. MT, FF-A, XM, and ME wrote study protocols and coordinated sample collection. KA, KK, and KB have verified the underlying data. All authors contributed to the article and approved the submitted version.

## Funding

We acknowledge the support from the Belgian Directorate-general Development Cooperation and Humanitarian Aid (DGD) for the Framework Agreement 4 project (2017-2021), the European Union’s Horizon 2020 research and innovation program, under the ZikaPLAN grant agreement 734584.4, the Flanders Innovation & Entrepreneurship (VLAIO) program for the Innovation mandate under the Grant Agreement number HBC.2018.0327 and the Fund for Scientific Research Flanders (FWO G054820N). FFA holds a PhD scholarship funded by the DGD.

## Conflict of Interest

The authors declare that the research was conducted in the absence of any commercial or financial relationships that could be construed as a potential conflict of interest.

## Supplementary Material

Supplementary Figure 1 Coverage of peptide library per virus. Peptides were plotted relative to polyprotein coordinates (start amino acid position) for DENV (P33478), ZIKV (A0A142I5B9) and YFV (P03314). Structural proteins: Capsid (C), Membrane (pr, M) and Envelope (E), and non-structural proteins: NS1, NS2A, NS2B, NS3, NS4A, NS4B and NS5 are color represented; each yellow dot represents a single unique peptide in the microarray.

Supplementary Figure 2 Antibodies from DENV-infected individuals target epitopes in linear peptides from DENV, ZIKV and YFV. Heatmaps depicting the relative number of peptides targeted by IgG and IgM Abs present in individual serum from endemic (n=10) and traveler patients (n=3) at the time samples were collected: T1 (acute), T2 (early convalescent), T3 (mid convalescent) and T4 (late convalescent). Each column represents a sample and each row represents a protein from the correspondent arbovirus. Samples from the negative subjects are the two last columns. The color intensity indicates the relative number of 15-mer peptides from the indicated arbovirus reacting against IgG and IgM antibodies.

Supplementary Figure 3 Longitudinal humoral responses to linear epitopes covering the ZIKV and YFV proteomes. The plots show the level of IgG **(A)** and IgM **(B)** Ab reactivity measured in fluorescence units relative to proteomic coordinates for ZIKV (Uniprot ref. A0A142I5B9) and YFV (Uniprot ref. P03314). Structural: Capsid (C), Membrane (pr, M) and Envelope (E), and non-structural proteins: NS1, NS2A, NS2B, NS3, NS4A, NS4B and NS5 are color represented; each dot represents the Ab binding to a single peptide in the microarray. In both **(A, B)** plots, the endemic group is at the top (four-time points), the traveler group at the center (two-time points) and the negatives at the bottom (one-time point). T1: acute (blue dots), T2: early convalescent (red dots), T3: mid convalescent (green dots) and T4: late convalescent (orange dots) samples. Responses from the endemic (E) and non-endemic (NE) negatives are represented in purple and cyan dots, respectively.

Supplementary Figure 4 Depth and breadth of the humoral response against ZIKV and YFV peptides. The plots show the Ab target regions (ATRs) for IgG **(A)** and IgM **(B)** aligned to the proteomic coordinates for ZIKV (Uniprot ref. A0A142I5B9) and YFV (Uniprot ref. P03314). Structural: Capsid (C), Membrane (pr, M) and Envelope (E), and non-structural proteins: NS1, NS2A, NS2B, NS3, NS4A, NS4B and NS5 are color represented. Each bar in the plot represents an ATR. The depth (different epitope variants recognized by the sera) of the Ab response is read vertically, the breadth (binding sites across the polyprotein) of the Ab response is read horizontally and the color intensity of each ATR indicates the magnitude of the Ab response, in terms of arbitrary fluorescence intensity units after Log2 transformation (ranging from 0 to 16). T1: acute, T2: early convalescent, T3: mid convalescent and T4: late convalescent (orange dots) samples. E: endemic and NE: non-endemic negative samples.

Supplementary Figure 5 Longitudinal IgG and IgM reactivity against NS3, NS5 DENV proteins and complete DENV proteome. The y axis indicates the fraction of endemic (n = 10) or traveler (n=3) patients reacting against peptides from NS3 **(A)**, NS5 **(B)** and DENV proteome **(C)** with either IgG (top, blue bars) or IgM (bottom, red bars) antibodies at the time points samples were collected. T1: acute, T2: early convalescent, T3: mid convalescent and T4: late convalescent (orange dots) samples. The x axis indicates the proteomic coordinates (amino acid start position) according to DENV Uniprot ref: P33478. The protein domains for NS3 and NS5 are color represented at the top of each plot.

Supplementary Figure 6 Frequency of IgG and IgM reactivity against ZIKV and YFV proteome. The y axis indicates the fraction of endemic (n = 10) or traveler (n=3) patients reacting against peptides from ZIKV **(A)** and YFV **(B)** proteomes with either IgG (top, blue bars) or IgM (bottom, red bars) antibodies at the time points samples were collected. T1: acute, T2: early convalescent, T3: mid convalescent and T4: late convalescent samples. The X axis indicates the proteomic coordinates (amino acid start position) according to ZIKV Uniprot ref: A0A142I5B9 and YFV Uniprot ref: P03314. The structural and non-structural proteins are color represented at the top of each plot.

Supplementary Figure 7 Longitudinal humoral response to ZIKV and YFV proteins. Each box represents the individual response of each patient through time in the endemic (from 1 to 10) and traveler (11 - 13) groups. The y axis indicates the Ab fold change per patient at the early-, mid- and late-convalescent samples, respect to the acute sample, colored by protein target. The x axis indicates the days when the convalescent samples were collected.

Supplementary Figure 8 Reactive epitopes from E and NS1 proteins among DENV, ZIKV and YFV flaviviruses. 15-mer peptides from DENV1-4, ZIKV and YFV targeted by IgG and IgM recognized by >5 endemic patients (or > two traveler patients) are indicated by horizontal colored lines. The upper row in each panel shows the proteomic coordinates according to the DENV Uniprot ref: P33478 and the domains of E and NS1 are color represented. E and NS1 amino acid sequences from DENV1-4, ZIKV and YFV were aligned and the conservation rate (percentage identity) between the amino acid positions is represented as green bar plots using a sliding window size of 15 amino acid positions. Red regions correspond to percentage identities below 30%. T1: acute, T2: early convalescent, T3: mid convalescent and T4: late convalescent samples. Regions highlighted in purple and green correspond to the most representative DENV-specific and Panflavi peptides, respectively. Cyan box corresponds to the location of the fusion loop.

Supplementary Figure 9 Reactive epitopes from NS3 and NS5 proteins among DENV, ZIKV and YFV flaviviruses. 15-mer peptides from DENV1-4, ZIKV and YFV targeted by IgG **(A)** and IgM **(B)** recognized by >5 endemic patients (or > two traveler patients) are indicated by horizontal colored lines. Proteomic coordinates and protein domains percentage identities are as in Supplementary Figure 8. T1: acute, T2: early convalescent, T3: mid convalescent and T4: late convalescent (orange dots) samples.

Supplementary Figure 10 Reactive epitopes from C, prM, NS2A, NS2B, NS4A and NS4B proteins among DENV, ZIKV and YFV flaviviruses. 15-mer peptides from DENV1-4, ZIKV and YFV targeted by IgG **(A)** and IgM **(B)** recognized by >5 endemic patients (or > two traveler patients) are indicated by horizontal colored lines. Proteomic coordinates and protein domains percentage identities are as in **Supplementary Figure S8**. T1: acute, T2: early convalescent, T3: mid convalescent and T4: late convalescent (orange dots) samples.

Supplementary Figure 11 Identification of DENV specific and flavivirus broadly immunogenic epitopes in NS3 and NS5 proteins. IgM and IgG targeted epitopes at the NS3 **(A)** and NS5 **(B)** proteins from DENV, ZIKV and YFV viruses, recognized by DENV patients were filtered out and mapped to the proteomic coordinates of DENV (Uniprot ref: P33478). Colored bars from light-yellow to orange represent immunodominant regions belonging to one DENV serotype. Red bars represent immunodominant regions belonging to more than one serotype here called PanDENV. Green bars represent immunodominant regions belonging to DENV and ZIKV or/and YFV here called PanFlavi. For both panels the protein domains and the percentage identities are as in **Supplementary Figure S8**. Red and green arrows are DENV-specific and PanFlavi peptides, respectively, which Abs responses are further detailed in **Supplementary Figure S13**.

Supplementary Figure 12 Identification of DENV specific and flavivirus broadly immunogenic epitopes in C, NS2A, NS2B, NS4A amd NS4B. IgM and IgG targeted epitopes at the E **(A)** and NS1 **(B)** proteins from DENV, ZIKV and YFV viruses, recognized by DENV patients were filtered out and mapped to the proteomic coordinates of DENV (Uniprot ref: P33478). Colored bars from light-yellow to orange represent immunodominant regions belonging to one DENV serotype. Red bars represent immunodominant regions belonging to more than one serotype here called PanDENV. Green bars represent immunodominant regions belonging to DENV and ZIKV or/and YFV here called PanFlavi. For both panels the protein domains and the percentage identities are as in **Supplementary Figure S8**. Red arrows are peptides which Abs responses are further detailed in **Supplementary Figure S13**. Purple and cyan arrows correspond to YFV and ZIKV peptides previously identified as ZIKV specific by Mishra et al. (31).

Supplementary Figure 13 Heat map showing DENV-specific and PanFlavi peptides targeted by IgM and IgG Abs. Heatmaps depicting the Ab reactivity from endemic patients, traveler patients and negative controls against each peptide. The left panel correspond to IgG selected peptides while the right panel to IgM selected peptides. Each column represents a sample. The heatmap color scale indicates the fluorescence intensity going from low (blue) to high (red). Each row represents a peptide from the correspondent protein and the coordinates of DENV (Uniprot ref: P33478).

Supplementary Figure 14 Heatmaps showing the humoral response against ZIKV and YFV ATRs. IgG (A) and IgM (B) responses from the endemic group against ZIKV and YFV ATRs were divided into patients with high neutralizing Abs (NAbs: (+) NTs, (+) PRNTs) titers or absent/low NAbs titers against ZIKV and YFV (see **Table 1**). T1 (acute), T2 (early convalescent), T3 (mid convalescent) and T4 (late convalescent) samples. Each column represents a sample. The heatmap color scale indicates the fluorescence intensity going from low (light yellow) to high (red). Each row represents an ATR.
