# Supplementary figures and images for: Dynamics of the Magnitude, Breadth and Depth of the Antibody Response at Epitope Level Following Dengue Infection
Source: Front Immunol. 2021 Jul 5;12:686691. doi: 10.3389/fimmu.2021.686691 (PMC8289389; doi:10.3389/fimmu.2021.686691)

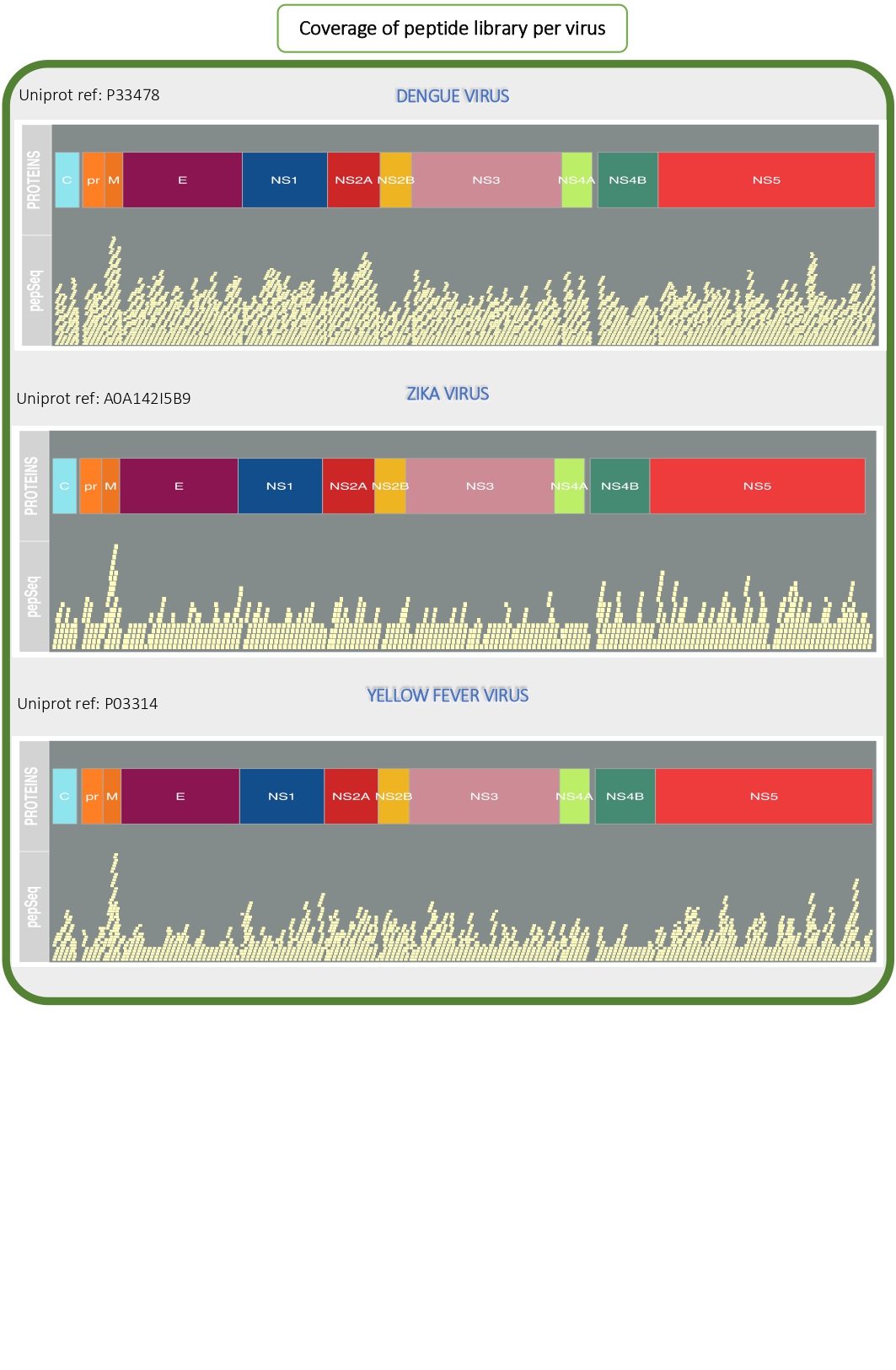

Supplement: Supplementary Figure 1 — Coverage of peptide library per virus. Peptides were plotted relative to polyprotein coordinates (start amino acid position) for DENV (P33478), ZIKV (A0A142I5B9) and YFV (P03314). Structural proteins: Capsid (C), Membrane (pr, M) and Envelope (E), and non-structural proteins: NS1, NS2A, NS2B, NS3, NS4A, NS4B and NS5 are color represented; each yellow dot represents a single unique peptide in the microarray. [file Image_1.jpeg]

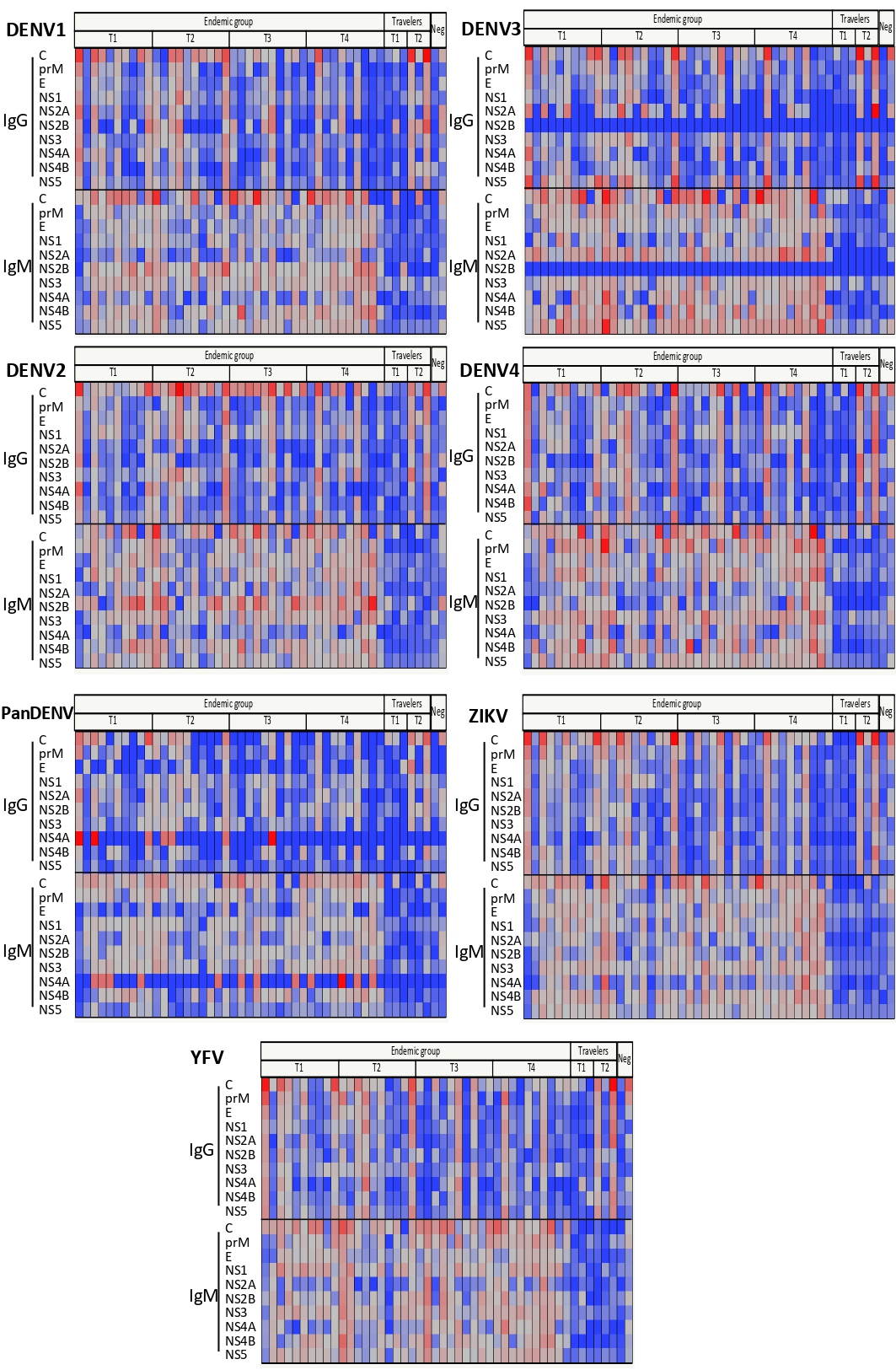

Supplement: Supplementary Figure 2 — Antibodies from DENV-infected individuals target epitopes in linear peptides from DENV, ZIKV and YFV. Heatmaps depicting the relative number of peptides targeted by IgG and IgM Abs present in individual serum from endemic (n=10) and traveler patients (n=3) at the time samples were collected: T1 (acute), T2 (early convalescent), T3 (mid convalescent) and T4 (late convalescent). Each column represents a sample and each row represents a protein from the correspondent arbovirus. Samples from the negative subjects are the two last columns. The color intensity indicates the relative number of 15-mer peptides from the indicated arbovirus reacting against IgG and IgM antibodies. [file Image_2.jpeg]

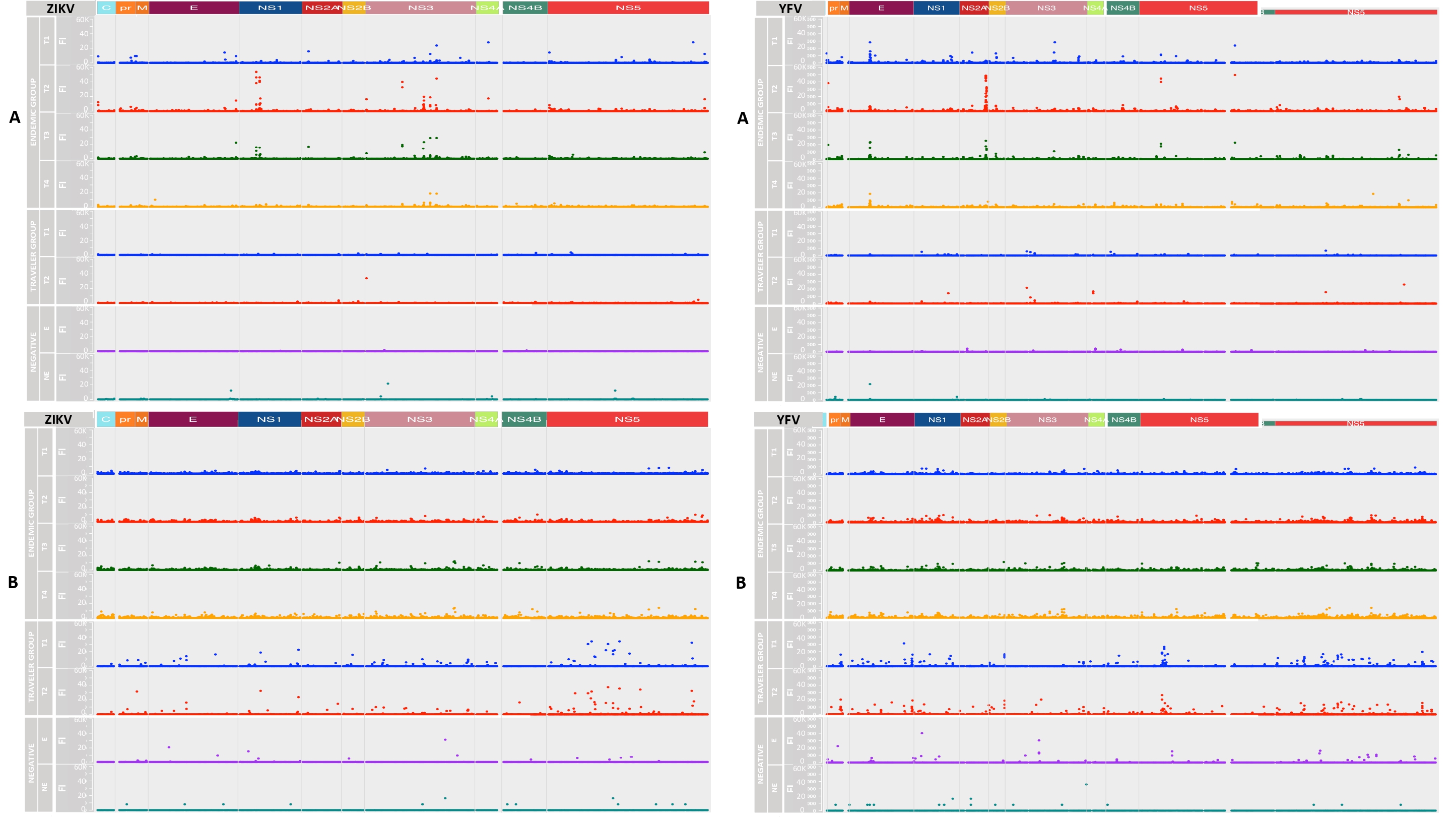

Supplement: Supplementary Figure 3 — Longitudinal humoral responses to linear epitopes covering the ZIKV and YFV proteomes. The plots show the level of IgG (A) and IgM (B) Ab reactivity measured in fluorescence units relative to proteomic coordinates for ZIKV (Uniprot ref. A0A142I5B9) and YFV (Uniprot ref. P03314). Structural: Capsid (C), Membrane (pr, M) and Envelope (E), and non-structural proteins: NS1, NS2A, NS2B, NS3, NS4A, NS4B and NS5 are color represented; each dot represents the Ab binding to a single peptide in the microarray. In both (A, B) plots, the endemic group is at the top (four-time points), the traveler group at the center (two-time points) and the negatives at the bottom (one-time point). T1: acute (blue dots), T2: early convalescent (red dots), T3: mid convalescent (green dots) and T4: late convalescent (orange dots) samples. Responses from the endemic (E) and non-endemic (NE) negatives are represented in purple and cyan dots, respectively. [file Image_3.jpeg]

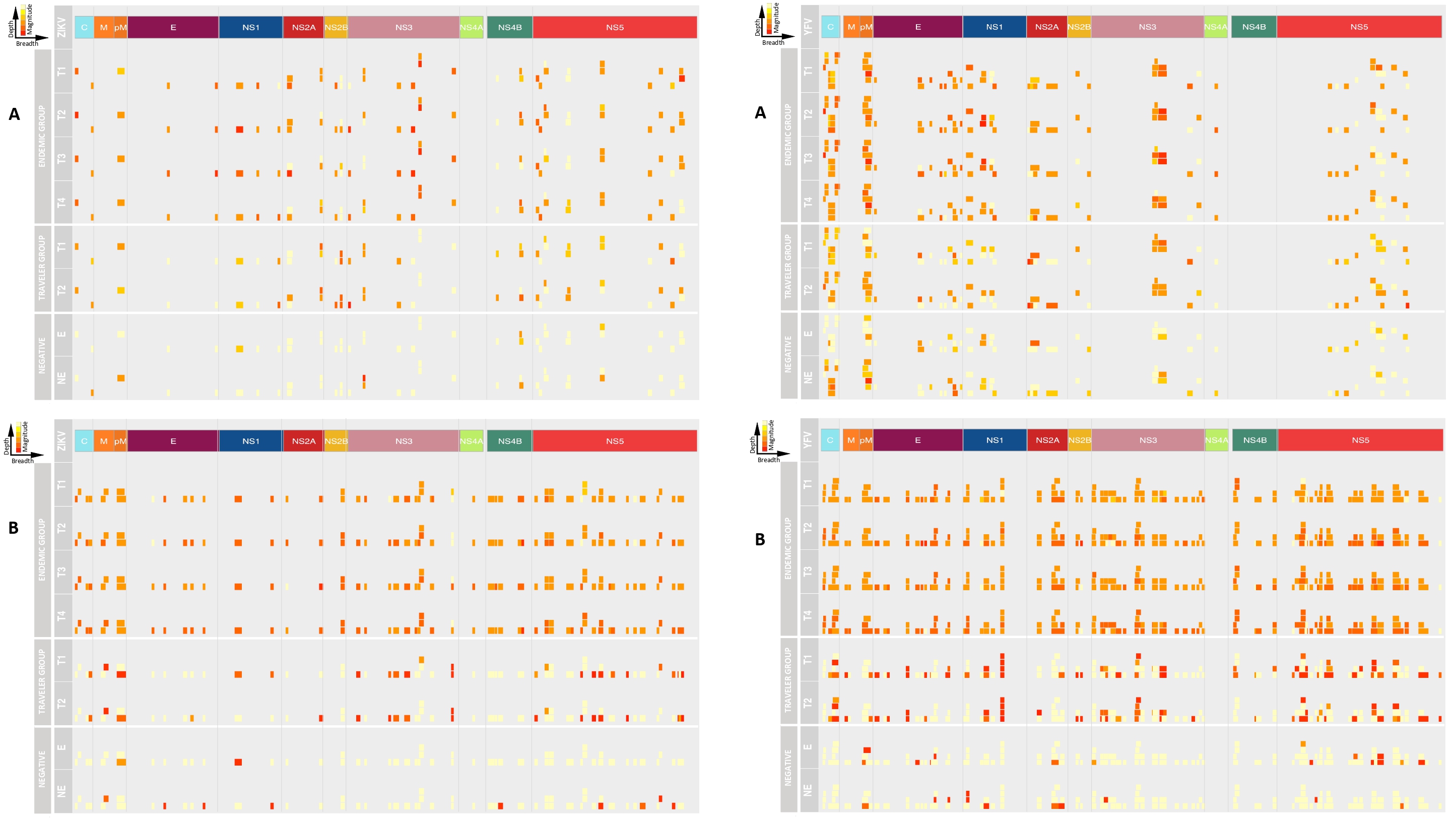

Supplement: Supplementary Figure 4 — Depth and breadth of the humoral response against ZIKV and YFV peptides. The plots show the Ab target regions (ATRs) for IgG (A) and IgM (B) aligned to the proteomic coordinates for ZIKV (Uniprot ref. A0A142I5B9) and YFV (Uniprot ref. P03314). Structural: Capsid (C), Membrane (pr, M) and Envelope (E), and non-structural proteins: NS1, NS2A, NS2B, NS3, NS4A, NS4B and NS5 are color represented. Each bar in the plot represents an ATR. The depth (different epitope variants recognized by the sera) of the Ab response is read vertically, the breadth (binding sites across the polyprotein) of the Ab response is read horizontally and the color intensity of each ATR indicates the magnitude of the Ab response, in terms of arbitrary fluorescence intensity units after Log2 transformation (ranging from 0 to 16). T1: acute, T2: early convalescent, T3: mid convalescent and T4: late convalescent (orange dots) samples. E: endemic and NE: non-endemic negative samples. [file Image_4.jpeg]

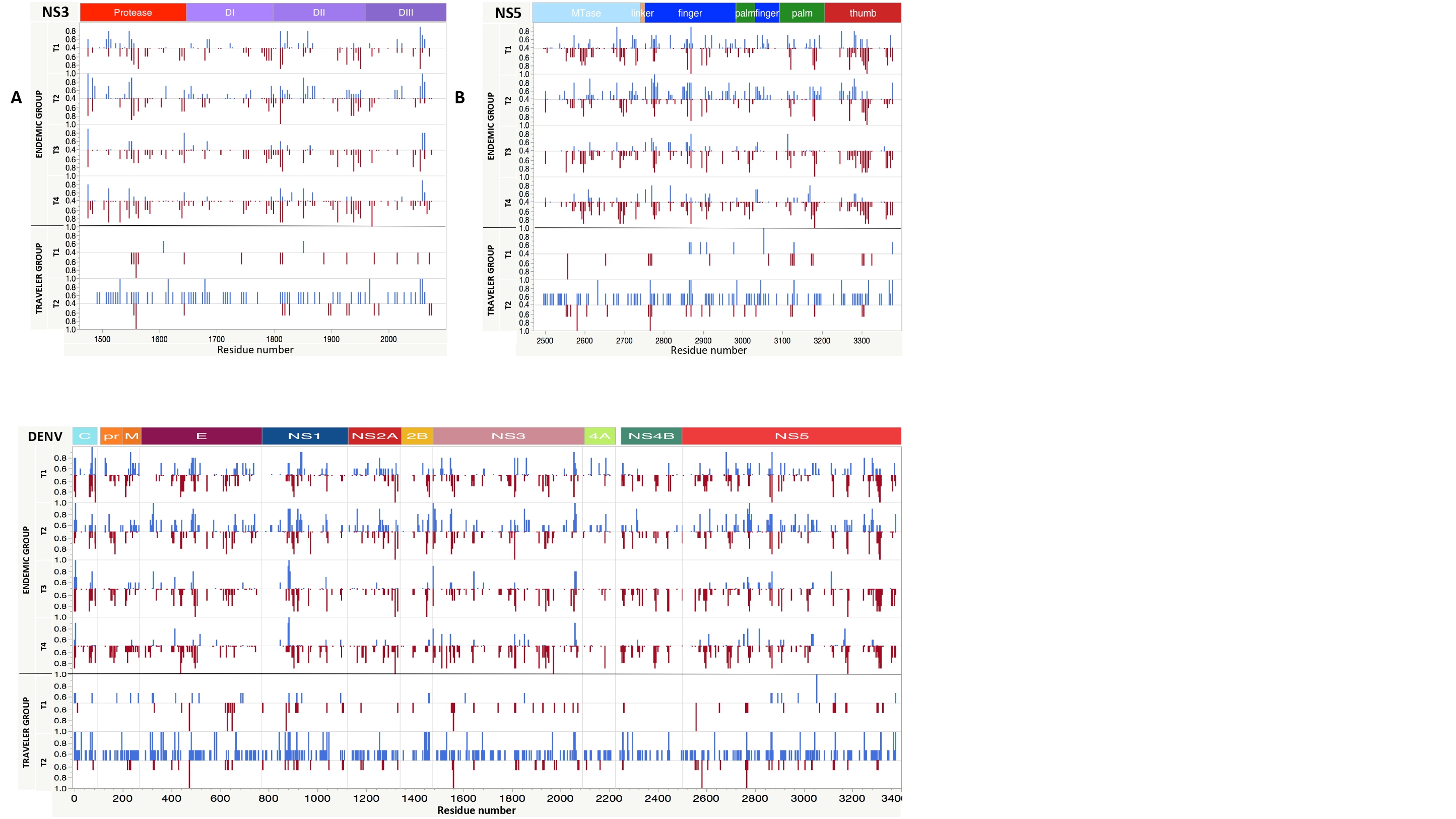

Supplement: Supplementary Figure 5 — Longitudinal IgG and IgM reactivity against NS3, NS5 DENV proteins and complete DENV proteome. The y axis indicates the fraction of endemic (n = 10) or traveler (n=3) patients reacting against peptides from NS3 (A), NS5 (B) and DENV proteome (C) with either IgG (top, blue bars) or IgM (bottom, red bars) antibodies at the time points samples were collected. T1: acute, T2: early convalescent, T3: mid convalescent and T4: late convalescent (orange dots) samples. The x axis indicates the proteomic coordinates (amino acid start position) according to DENV Uniprot ref: P33478. The protein domains for NS3 and NS5 are color represented at the top of each plot. [file Image_5.jpeg]

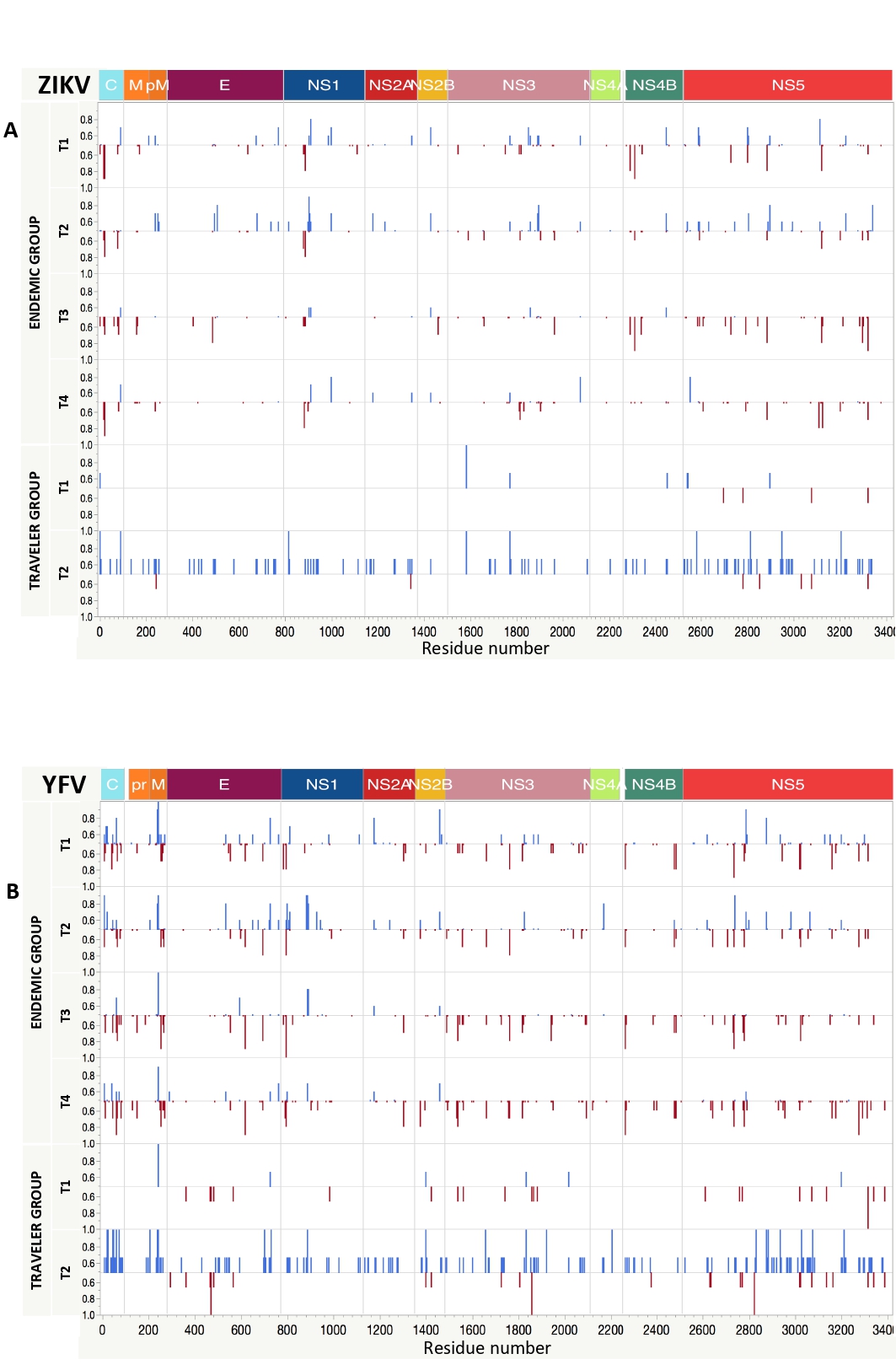

Supplement: Supplementary Figure 6 — Frequency of IgG and IgM reactivity against ZIKV and YFV proteome. The y axis indicates the fraction of endemic (n = 10) or traveler (n=3) patients reacting against peptides from ZIKV (A) and YFV (B) proteomes with either IgG (top, blue bars) or IgM (bottom, red bars) antibodies at the time points samples were collected. T1: acute, T2: early convalescent, T3: mid convalescent and T4: late convalescent samples. The X axis indicates the proteomic coordinates (amino acid start position) according to ZIKV Uniprot ref: A0A142I5B9 and YFV Uniprot ref: P03314. The structural and non-structural proteins are color represented at the top of each plot. [file Image_6.jpeg]

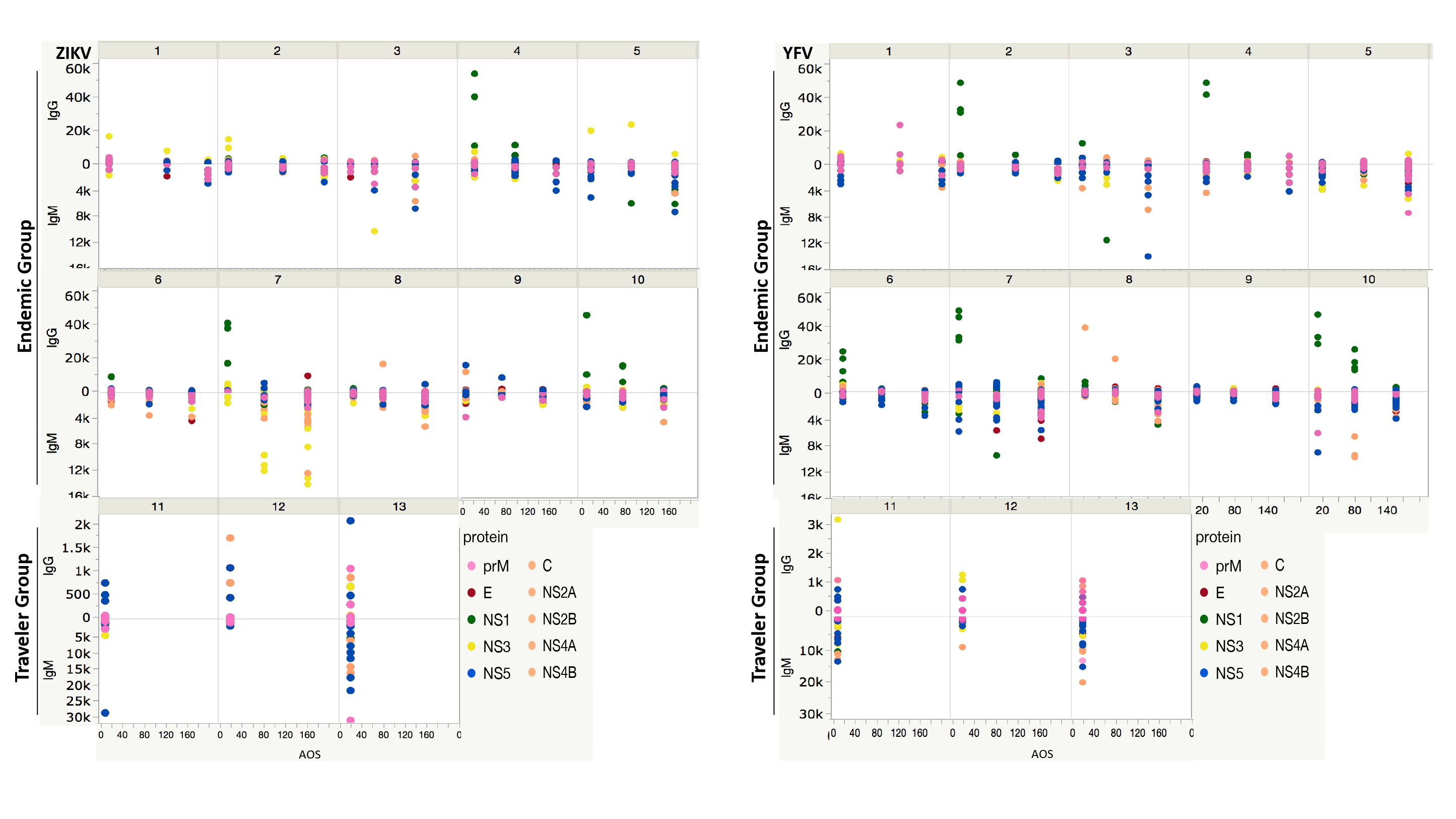

Supplement: Supplementary Figure 7 — Longitudinal humoral response to ZIKV and YFV proteins. Each box represents the individual response of each patient through time in the endemic (from 1 to 10) and traveler (11 - 13) groups. The y axis indicates the Ab fold change per patient at the early-, mid- and late-convalescent samples, respect to the acute sample, colored by protein target. The x axis indicates the days when the convalescent samples were collected. [file Image_7.jpeg]

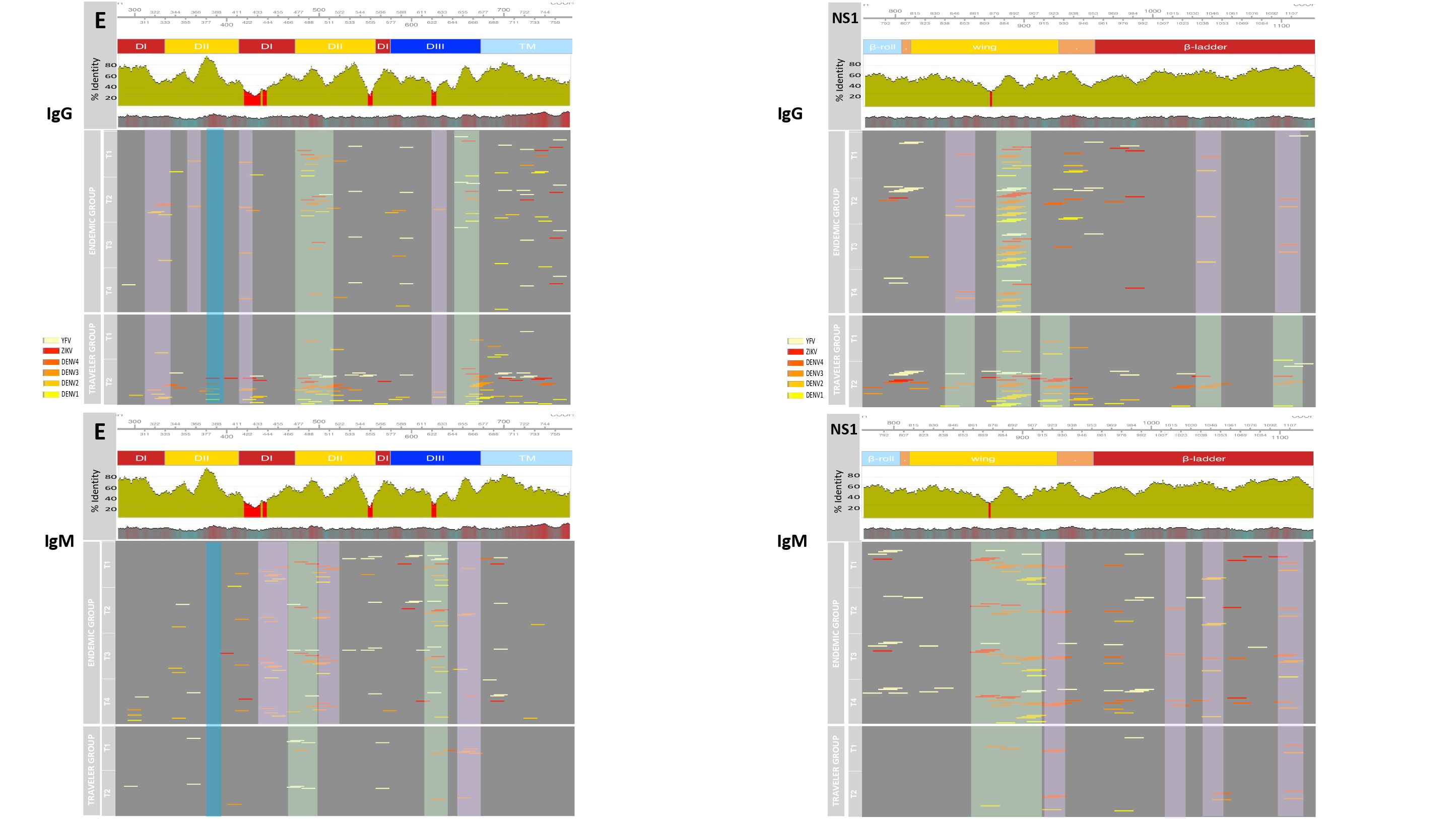

Supplement: Supplementary Figure 8 — Reactive epitopes from E and NS1 proteins among DENV, ZIKV and YFV flaviviruses. 15-mer peptides from DENV1-4, ZIKV and YFV targeted by IgG and IgM recognized by >5 endemic patients (or > two traveler patients) are indicated by horizontal colored lines. The upper row in each panel shows the proteomic coordinates according to the DENV Uniprot ref: P33478 and the domains of E and NS1 are color represented. E and NS1 amino acid sequences from DENV1-4, ZIKV and YFV were aligned and the conservation rate (percentage identity) between the amino acid positions is represented as green bar plots using a sliding window size of 15 amino acid positions. Red regions correspond to percentage identities below 30%. T1: acute, T2: early convalescent, T3: mid convalescent and T4: late convalescent samples. Regions highlighted in purple and green correspond to the most representative DENV-specific and Panflavi peptides, respectively. Cyan box corresponds to the location of the fusion loop. [file Image_8.jpeg]

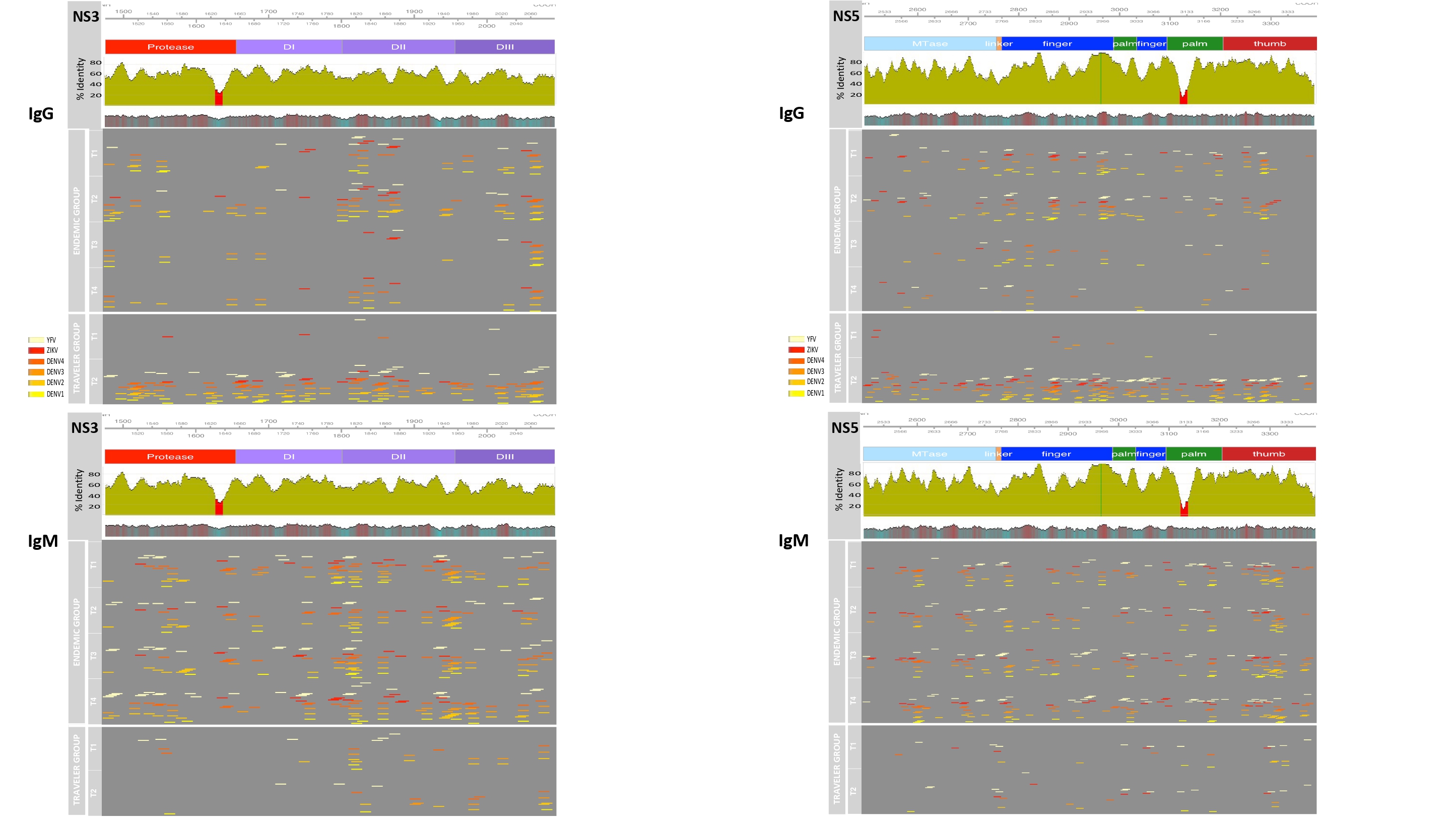

Supplement: Supplementary Figure 9 — Reactive epitopes from NS3 and NS5 proteins among DENV, ZIKV and YFV flaviviruses. 15-mer peptides from DENV1-4, ZIKV and YFV targeted by IgG (A) and IgM (B) recognized by >5 endemic patients (or > two traveler patients) are indicated by horizontal colored lines. Proteomic coordinates and protein domains percentage identities are as in Supplementary Figure 8. T1: acute, T2: early convalescent, T3: mid convalescent and T4: late convalescent (orange dots) samples. [file Image_9.jpeg]

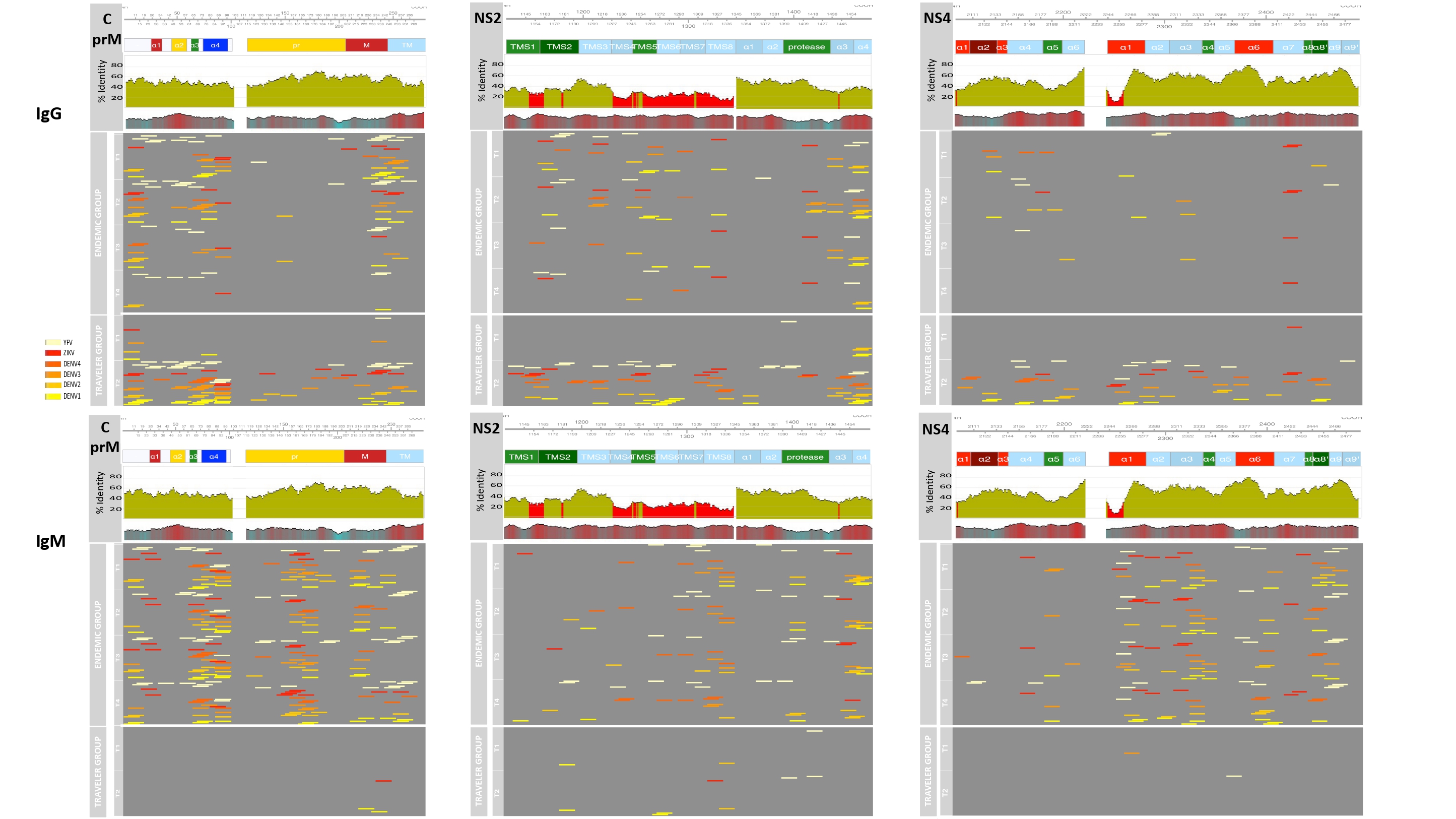

Supplement: Supplementary Figure 10 — Reactive epitopes from C, prM, NS2A, NS2B, NS4A and NS4B proteins among DENV, ZIKV and YFV flaviviruses. 15-mer peptides from DENV1-4, ZIKV and YFV targeted by IgG (A) and IgM (B) recognized by >5 endemic patients (or > two traveler patients) are indicated by horizontal colored lines. Proteomic coordinates and protein domains percentage identities are as in Supplementary Figure S8 . T1: acute, T2: early convalescent, T3: mid convalescent and T4: late convalescent (orange dots) samples. [file Image_10.jpeg]

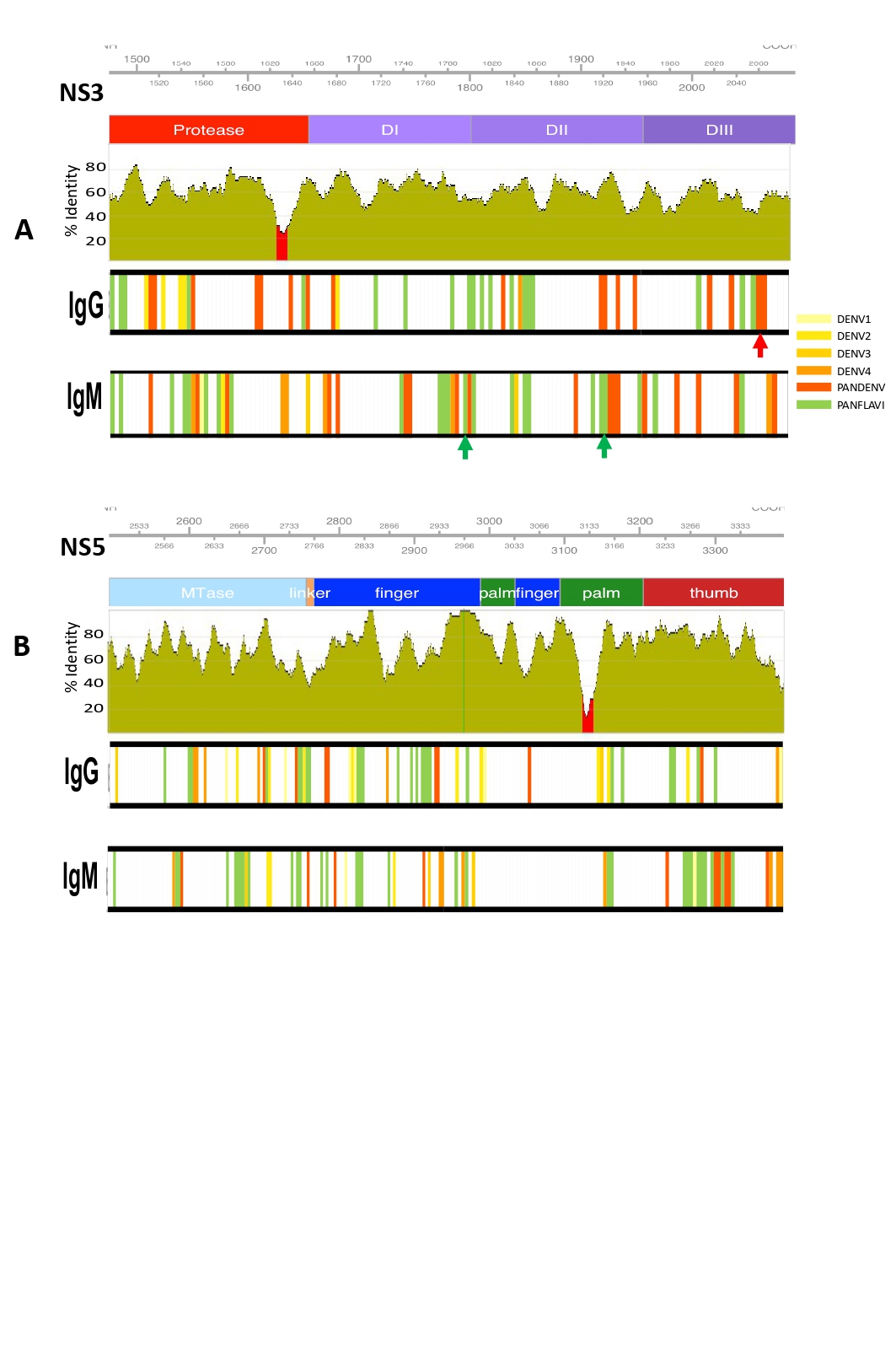

Supplement: Supplementary Figure 11 — Identification of DENV specific and flavivirus broadly immunogenic epitopes in NS3 and NS5 proteins. IgM and IgG targeted epitopes at the NS3 (A) and NS5 (B) proteins from DENV, ZIKV and YFV viruses, recognized by DENV patients were filtered out and mapped to the proteomic coordinates of DENV (Uniprot ref: P33478). Colored bars from light-yellow to orange represent immunodominant regions belonging to one DENV serotype. Red bars represent immunodominant regions belonging to more than one serotype here called PanDENV. Green bars represent immunodominant regions belonging to DENV and ZIKV or/and YFV here called PanFlavi. For both panels the protein domains and the percentage identities are as in Supplementary Figure S8 . Red and green arrows are DENV-specific and PanFlavi peptides, respectively, which Abs responses are further detailed in Supplementary Figure S13 . [file Image_11.jpeg]

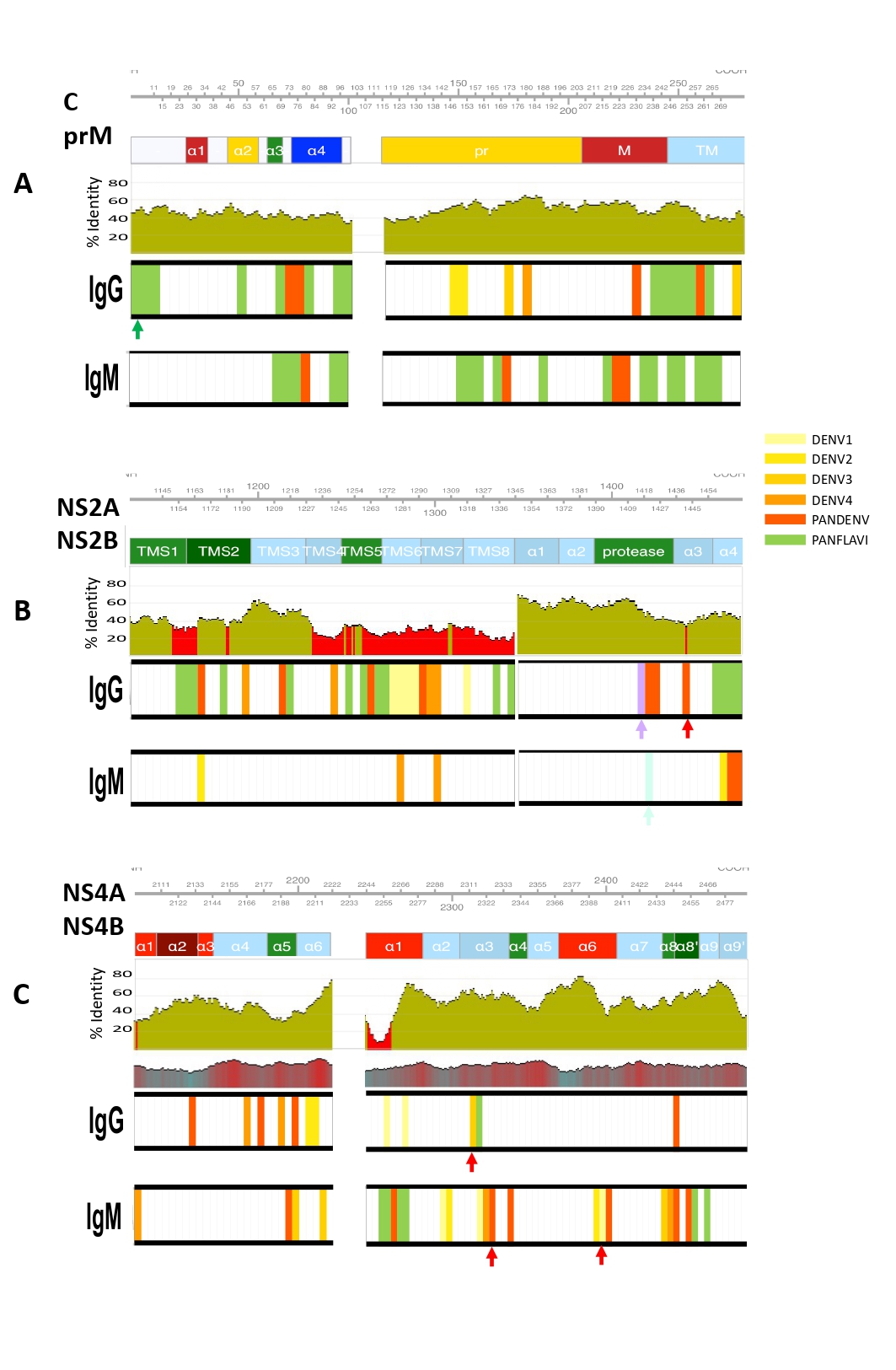

Supplement: Supplementary Figure 12 — Identification of DENV specific and flavivirus broadly immunogenic epitopes in C, NS2A, NS2B, NS4A amd NS4B. IgM and IgG targeted epitopes at the E (A) and NS1 (B) proteins from DENV, ZIKV and YFV viruses, recognized by DENV patients were filtered out and mapped to the proteomic coordinates of DENV (Uniprot ref: P33478). Colored bars from light-yellow to orange represent immunodominant regions belonging to one DENV serotype. Red bars represent immunodominant regions belonging to more than one serotype here called PanDENV. Green bars represent immunodominant regions belonging to DENV and ZIKV or/and YFV here called PanFlavi. For both panels the protein domains and the percentage identities are as in Supplementary Figure S8 . Red arrows are peptides which Abs responses are further detailed in Supplementary Figure S13 . Purple and cyan arrows correspond to YFV and ZIKV peptides previously identified as ZIKV specific by Mishra et al. (31). [file Image_12.jpeg]

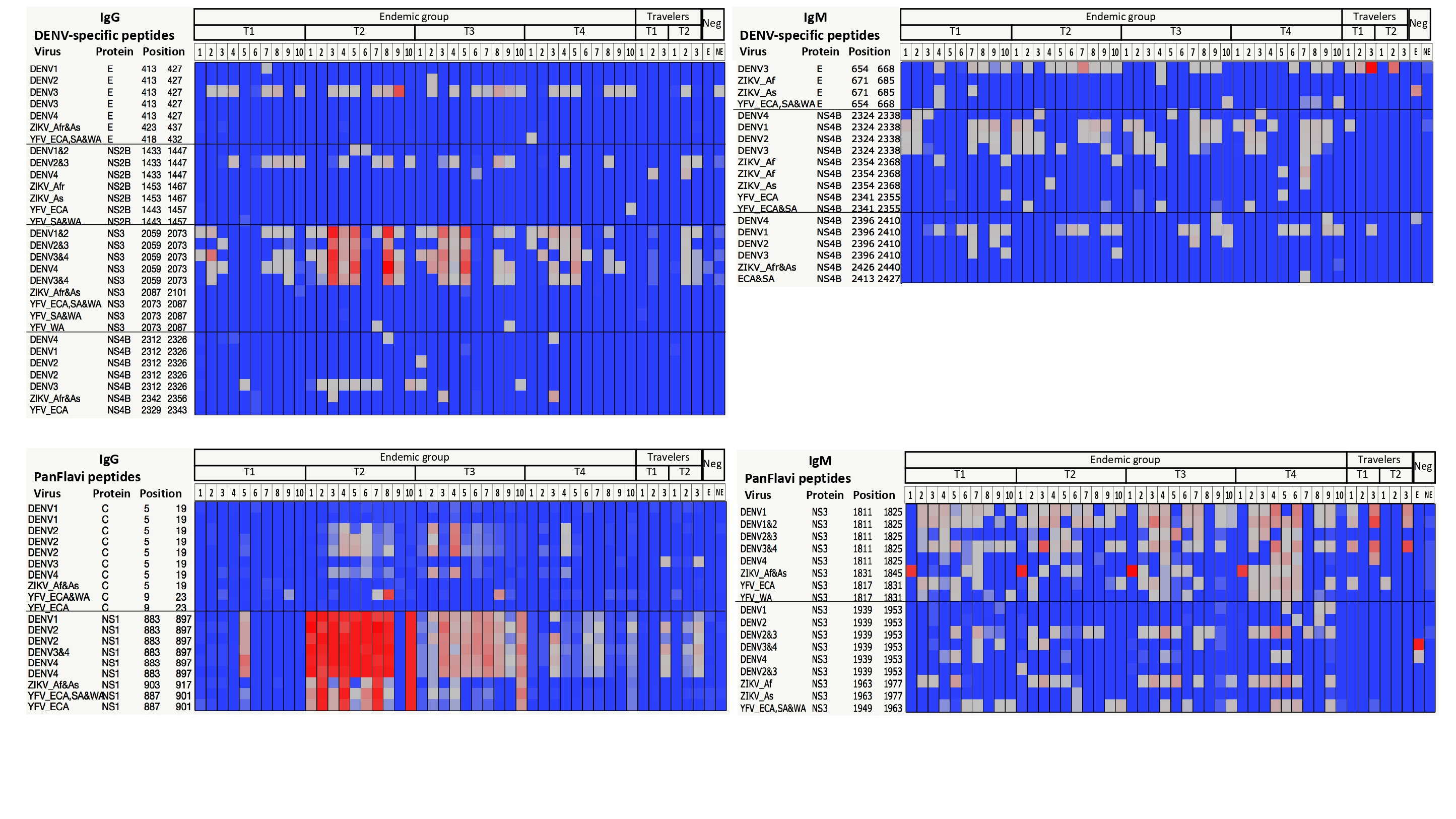

Supplement: Supplementary Figure 13 — Heat map showing DENV-specific and PanFlavi peptides targeted by IgM and IgG Abs. Heatmaps depicting the Ab reactivity from endemic patients, traveler patients and negative controls against each peptide. The left panel correspond to IgG selected peptides while the right panel to IgM selected peptides. Each column represents a sample. The heatmap color scale indicates the fluorescence intensity going from low (blue) to high (red). Each row represents a peptide from the correspondent protein and the coordinates of DENV (Uniprot ref: P33478). [file Image_13.jpeg]

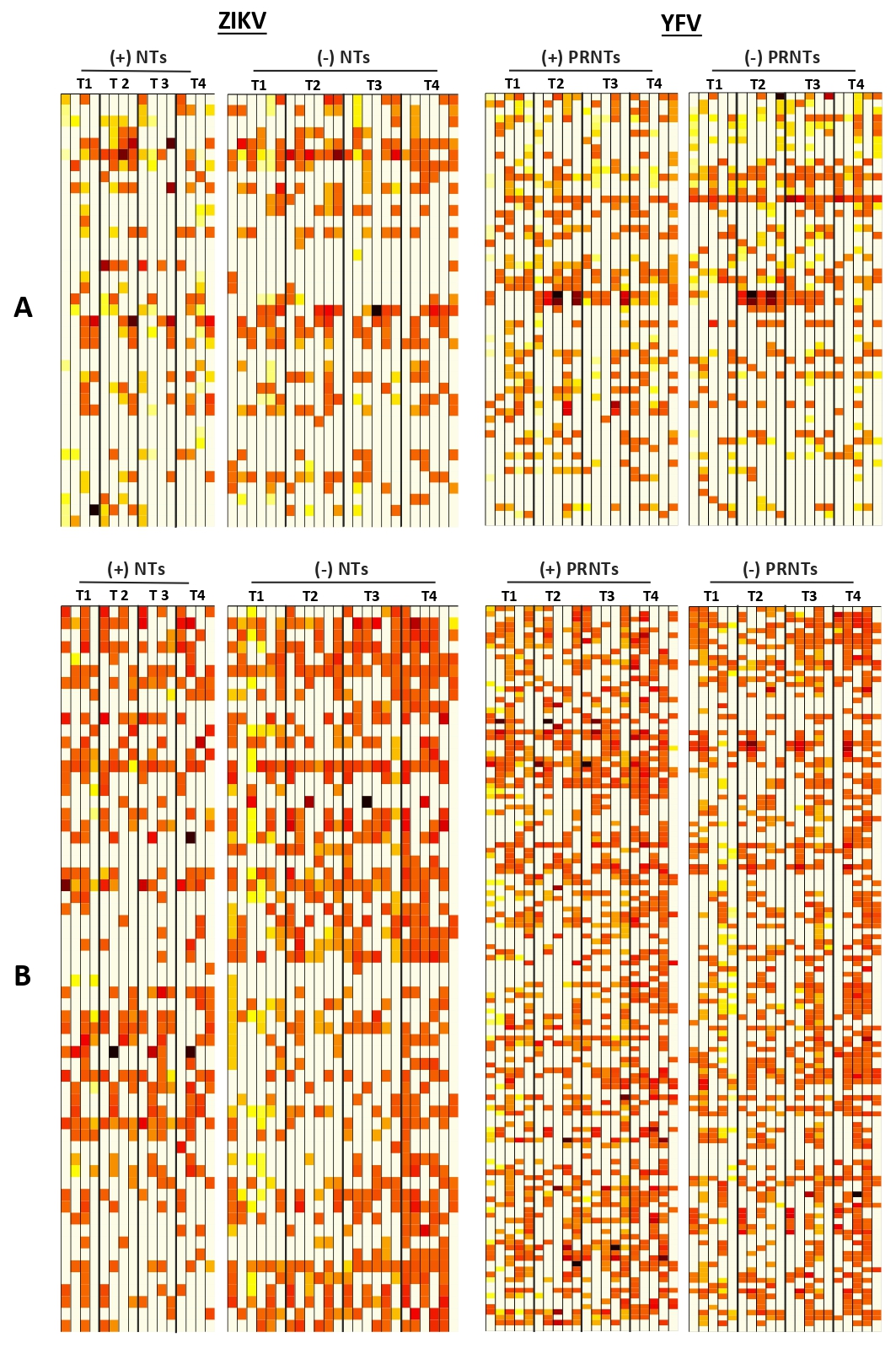

Supplement: Supplementary Figure 14 — Heatmaps showing the humoral response against ZIKV and YFV ATRs. IgG (A) and IgM (B) responses from the endemic group against ZIKV and YFV ATRs were divided into patients with high neutralizing Abs (NAbs: (+) NTs, (+) PRNTs) titers or absent/low NAbs titers against ZIKV and YFV (see Table 1 ). T1 (acute), T2 (early convalescent), T3 (mid convalescent) and T4 (late convalescent) samples. Each column represents a sample. The heatmap color scale indicates the fluorescence intensity going from low (light yellow) to high (red). Each row represents an ATR. [file Image_14.jpeg]
